# Repeatable, low-drift recordings in behaving nonhuman primates using flexible microelectrodes

**DOI:** 10.1038/s41467-026-77421-3

**Published:** 2026-09-09

**Authors:** Daniel P. Woods, Grace M. Adams, Rana Mozumder, Wenhao Dang, Andrew Y. Chen, Maxime Chevée, Christos Constantinidis, Daniel L. Gonzales

**Affiliations:** 1 Department of Biomedical Engineering, Vanderbilt University, Nashville, TN USA; 2 Program in Neuroscience, Vanderbilt University, Nashville, TN USA; 3 Department of Pharmacology, Vanderbilt University, Nashville, TN USA; 4 Department of Ophthalmology and Visual Science, Vanderbilt University Medical Center, Nashville, TN USA; 5 Vanderbilt Brain Institute, Vanderbilt University, Nashville, TN USA

**Keywords:** Neuroscience, Biomedical engineering

## Abstract

Neurophysiological recordings from nonhuman primates rely on rigid microelectrode arrays whose mechanical mismatch with brain tissue causes neuronal drift and unstable single-unit recordings. Here we present a flexible microelectrode platform for repeated recordings in awake rhesus macaques. We fabricated a 32-channel, 7-μm-thick Parylene-C array and developed a telescopic insertion method using concentric guide tubes and a retractable microwire shuttle to repeatedly deliver free-floating arrays through intact dura using standard recording chambers. Across two animals, we optimized the electrode geometry and insertion to achieve an 80% single-unit recording success rate. During an oculomotor delayed response task, we recorded stable task-responsive neurons from prefrontal and posterior parietal cortex. Compared with rigid probes in the same animals and recording chambers, flexible arrays achieved a comparable single-unit yield while reducing drift from hundreds to tens of micrometers. Our work establishes flexible microelectrodes as a practical and high-performing technology for primate neuroscience.

## Abstract (web summary)

Stable single-neuron recordings are essential for understanding brain function and enabling future neurotechnologies. Here, the authors demonstrate that flexible polymer microelectrodes provide repeatable recordings in awake nonhuman primates with comparable yield and reduced electrode drift.

## Introduction

High-fidelity neurophysiological recordings obtained from nonhuman primates (NHPs) are essential for mapping the circuit dynamics of cognition, yet current technologies face fundamental limitations that compromise recording quality and long-term stability. Multi-site recordings in these large-brained animals have traditionally relied on devices constructed from rigid materials. For example, steel microelectrode arrays such as Plexon S/U/V-probes have been a staple of NHP neurophysiology for decades^1,2^ and silicon Utah Arrays have provided the technological basis for high performing brain-computer interfaces^3–6^. More recently, high-density silicon probes such as Neuropixels have revolutionized the field by recording from hundreds of neurons simultaneously with high spatial resolution^7,8^. Despite these technological advances, all rigid electrode technologies share a fundamental limitation: a mechanical mismatch between stiff materials (Young’s moduli of approximately 200 GPa for steel and silicon) and soft brain tissue (approximately 1–10 kPa)^9,10^. In particular, brain micromotion due to respiration, cardiac rhythm, and cerebrospinal fluid (CSF) flow causes shearing against the inserted neural interface, resulting in the physiological drift of neurons relative to the fixed position of microelectrodes^11,12^. In large animals and humans, this motion can be on the order of hundreds of microns^13,14^. Even during acute recordings lasting only a few hours, drift compromises the ability to track individual neural units and complicates data analysis^15,16^. For chronic implants, drift leads to large fluctuations in the day-to-day yield of unit activity^17^.

To mitigate these challenges, flexible penetrating electrodes have emerged as a promising alternative to rigid probes. Materials such as polyimide, Parylene-C, and SU-8 provide enhanced mechanical compliance with brain tissue^18^. In rodents, there is robust evidence that flexible thin-film electrodes integrate with neural tissue and provide stable recordings after implantation^19–26^. However, these technologies have been slow to be adopted for NHPs. The challenge of neurophysiology is magnified in large animals by intensive, high-risk surgeries and thick dura mater that must be removed or penetrated for intracranial recordings. Surgical removal of the dura carries risks including CSF leakage, inflammation, and infection^27^. Dura penetration is not without risk as well. Brittle probes can break or become damaged during insertion. Even rigid electrodes such as silicon Neuropixels and steel Plexon probes often require large guide tubes that penetrate through dura and allow for microelectrode array insertion^8,28^. Flexible electrodes face an even greater challenge. Their mechanical compliance—the very property that makes them appealing for tissue integration—renders them difficult to deploy as free-floating arrays in brain tissue. Current insertion methods for flexible electrodes require complex, customized combinations of temporary stiffening shuttles and biodegradable coatings, often in conjunction with complete dura removal during open skull surgical procedures^10,29,30^. Compared to well-established techniques in NHPs for the repeated insertion of rigid arrays through chronic recording chambers, this additional complexity has made the practical advantages of flexible electrodes unclear. Therefore, while flexible electrodes theoretically offer a new frontier of low-footprint, highly stable recordings in NHPs, the field lacks clear demonstrations that the benefits outweigh the substantial challenges.

Existing studies using flexible neural interfaces in NHPs indeed suggest that this approach offers a stable interface to the brain^31–39^. Chronic polyimide implants have been able to record local field potentials^36^ and spiking activity^31,35,38^, with some instances of reliable single-unit tracking across recording sessions^38^. Proof-of-concept recordings with flexible geometries and materials have also been performed in NHPs in acute settings, typically in anesthetized animals^32–34,37^. In the vast majority of both chronic and acute cases, these implantations were conducted following a new craniotomy and dura removal. A small number of studies have inserted flexible microelectrode arrays through traditional chronic recording chambers without requiring a new craniotomy^34,39^. In these instances, the stiffening shuttle used for insertion was either not removed or unable to be reliably removed, therefore impeding the full potential of the flexible array. Together, these studies highlight the promise of emerging capabilities such as wireless recording^36^ and increasingly large-scale, high-density flexible arrays^34,38^, but also underscore that flexible neural interfaces in NHPs remain a nascent and technically constrained field.

We identify two key areas that need further development to usher in an era of ultra-stable, single-neuron recordings in NHPs with flexible arrays. First, we must establish robust, low-risk implantation protocols that can be easily adopted by NHP research groups. This requires approaches that circumvent the need for a new craniotomy prior to every session and have a high likelihood of successful recording. While several insertion techniques have been demonstrated for NHPs^34–36,38,39^, studies typically report only a few recordings and rarely provide statistics regarding the number of recording attempts and successes. Without quantitative data on these surgical workflows, the barrier to entry for NHP neurophysiology groups remains high. Second, we need direct, quantitative demonstrations comparing the recording stability of rigid and flexible microelectrodes. While flexible probes are hypothesized to reduce drift by complying with brain micromotion, there are no side-by-side comparisons between flexible and rigid arrays to validate this. The current lack of data in these two areas of reliable insertion and validated stability obscures the advantages of flexible electrodes compared to established technology and hinders their adoption by the broader NHP community.

Here, we present a comprehensive approach to these challenges by detailing a system for non-surgical, repeatable, and highly stable single-unit recordings in behaving NHPs using flexible microelectrodes. We introduce a telescopic insertion method that combines a conventional guide tube with a retractable shuttle mechanism for deploying free-floating arrays through intact dura. Our non-surgical insertion occurs through standard chronic recording chambers in awake animals without the need for a new craniotomy and dura resection, allowing for daily recording sessions for multiple weeks. This approach also mirrors conventional methods for NHP recordings, therefore lowering the barrier for adoption. We performed acute recordings in prefrontal cortex (PFC) and posterior parietal cortex (PPC) in actively behaving NHPs performing a working memory task. We report 21 total recordings, far surpassing similar studies with flexible arrays. After refining our electrodes and methodology, we achieved a recording success rate in 80% of sessions. Furthermore, in 18 recordings we also tracked animals’ behavior and observed high-yield, single units with high signal-to-noise ratios that were associated with task-relevant variables. Importantly, our implantation approach allowed us to systematically quantify the stability and drift associated with free-floating flexible probes in the NHP cortex. When compared to rigid microelectrode arrays, we found that flexible electrodes dramatically reduced the drift of single-unit recordings. By answering the critical insertion and stability questions that have hindered their translation to large animal models, our research paves the way for reliable deployment of flexible neural interfaces in NHPs.

## Results

### Fabrication of mechanically stable flexible arrays

**Fig. 1:**
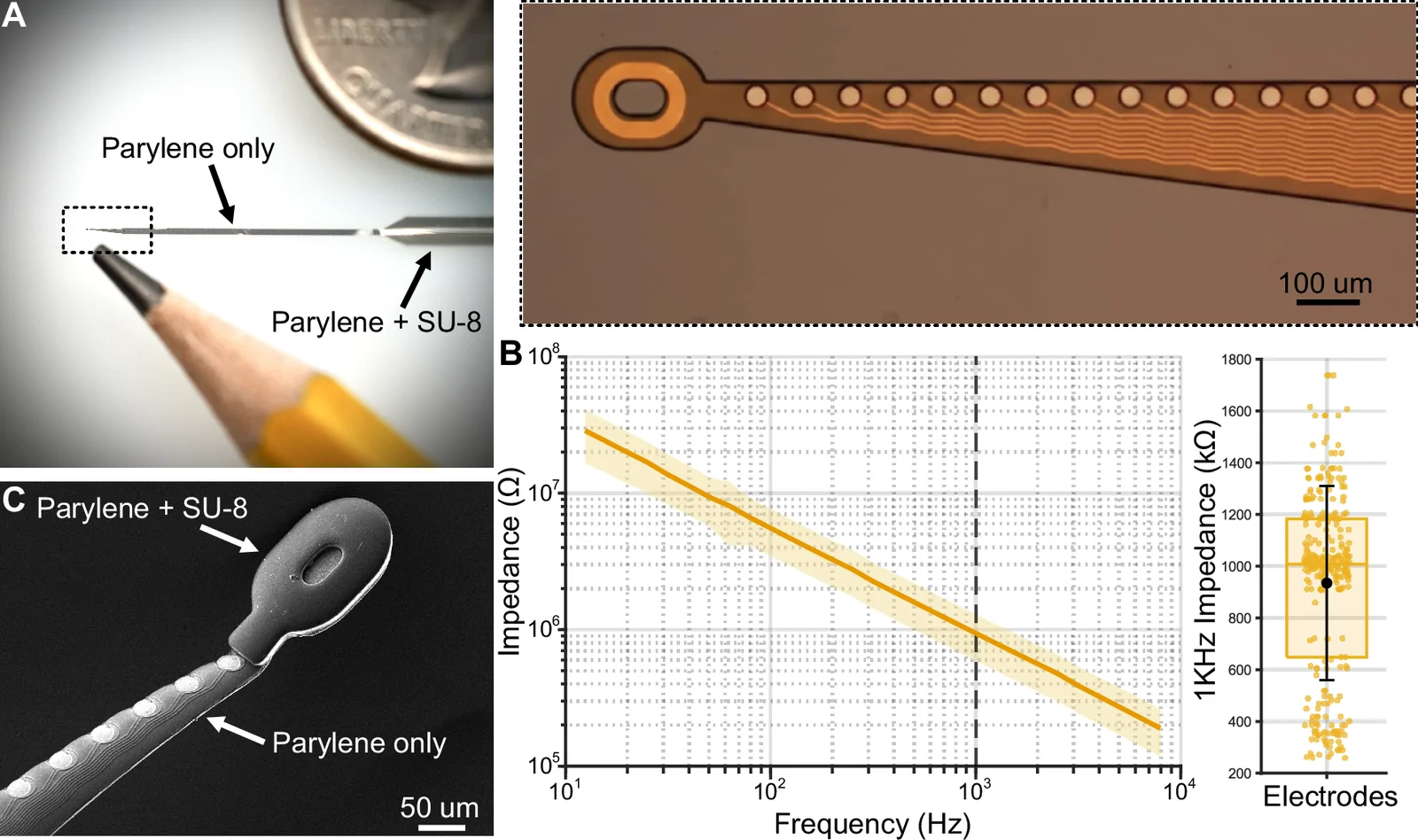
Flexible Parylene-C microelectrode arrays for NHP recordings. **A** Photograph of a flexible, Parylene-C probe. Inset shows microscope image of the microelectrode array. **B** Electrochemical impedance measurements of the Pt microelectrodes (*n* = 307 electrodes across 10 fabricated arrays). The solid line shows the mean impedance and the shaded region indicates ±1 standard deviation. Scatter plot shows the mean and standard deviation of the impedance at 1 kHz. For box plot, the center line indicates the median, the box bounds indicate the 25th and 75th percentiles, whiskers extend to the most extreme data points within 1.5× the interquartile range (IQR), and points beyond the whiskers are plotted individually as outliers. **C** Scanning electron micrograph of the microelectrode array and probe tip. Top arrow indicates probe tip layered with 10 µm SU-8 layer reinforcing the 7 µm-thick Parylene-C through-hole. Bottom arrow indicates recording shank that has no SU-8 top layer.

To achieve both flexibility and mechanical robustness, we designed custom Parylene-C arrays that strategically balance these competing demands (Fig. 1). We fabricated flexible arrays using a standard combination of chemical vapor deposition of Parylene-C, photolithography, and thin-film sputter deposition (Supplementary Fig. 1, Methods)^40^. Probes consisted of a 32-channel linear array spanning approximately 1 mm (Fig. 1A). We specifically designed and evaluated two sets of Pt microelectrode arrays, one with a checkerboard distribution of electrodes, and the other with a linear array at the probe edge. Each recording site had a diameter of 30 µm, yielding approximately 706 µm² contact area and mean electrochemical impedance at 1 kHz of approximately 935 ± 21 kΩ (Fig. 1B), with no significant association between the electrochemical impedance and recording site position along the arrays (Supplementary Fig. 2). Comparatively, we measured the 1 kHz impedance for two commercial rigid arrays, 32-channel Plexon S/V probes and 128-channel Diagnostic BioChips (DBC) Deep Array probes, which had average values of 455 ± 19 kΩ and 378 ± 13 kΩ, respectively (Supplementary Fig. 2).

Throughout the design process, we balanced the tradeoffs of ultra-flexibility with the mechanical robustness for reliable implantation in large-brained animals. The probes had an average Parylene-C thickness of 7 µm and a width of 300 µm, tapering down to 50 µm at the probe tip. To enhance mechanical stability, we added an additional 10 µm layer of SU-8 that covered the probe backend (connectors and ribbon, Fig. 1A). Similar to previous studies^41^, we found that this dramatically reduced probe curling and eased the process of preparing probes for recordings. We also strategically placed a through-hole at the probe tip for facilitating implantation via a microwire shuttle^21,38,39^ and surrounded it with SU-8 to reduce the likelihood of tearing during insertion (Fig. 1C). These design features produced probes that were thin and compliant enough to move with brain tissue, yet robust enough to withstand the mechanical stresses of insertion through intact dura in large animals.

### Telescopic delivery of flexible electrodes in awake NHPs

**Fig. 2:**
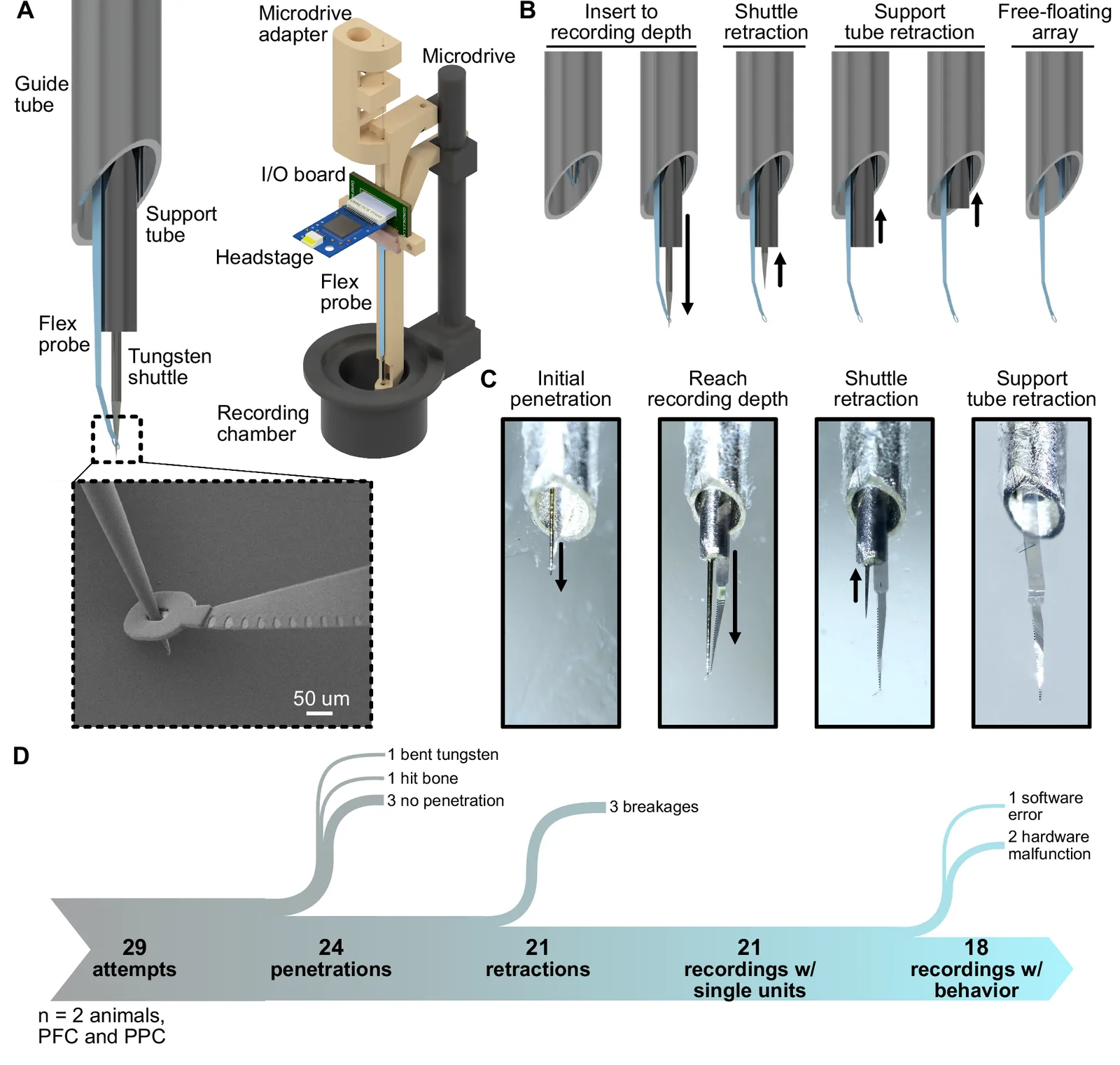
Telescopic insertion of flexible arrays. **A** 3D model of the full insertion system and telescoping mechanism for implanting flexible probes. Headstage rendered using the Intan C3313 32-channel 3D model provided by Intan Technologies. The inset shows an SEM of the sharpened tungsten shuttle threading the Parylene-C through-hole. Contacts are shown facing inward for visualization. During recordings, the probe was oriented with the contacts facing the tissue. **B** 3D model outlining the step-by-step process of implanting flexible electrodes using telescopic insertion. **C** Example insertion of a flexible microelectrode array in agar. **D** Flow chart displaying the overall implantation outcomes and failure modes across all recording attempts. Outcomes are shown for 29 implantation attempts across two animals.

The key technical challenges for flexible electrodes in NHPs are delivering them through thick dura and removing the stiffening shuttle. We addressed this through a two-stage telescopic implantation approach that reliably deployed our flexible electrodes into the awake NHP brain without the need for a new craniotomy or dura removal (Fig. 2, Methods). Our approach parallels conventional NHP neurophysiological recordings and incorporates a 19 G steel guide tube for penetration through dura/pia mater (Fig. 2A). To guide the flexible microelectrode through the guide tube and to the recording depth, our system relied on a sharpened tungsten microwire threaded through the tip of our flexible probe (Fig. 2A), mirroring previous methods in rodents^21^ and NHPs^38,39^. However, when this technique has been employed for large-brained animals, there are reports of an inability to retract the shuttle^39^. Often, detachment of the microwire and flexible array is unreliable, and both the shuttle and microelectrode exit tissue during retraction. To overcome this challenge, we drew inspiration from previous studies that temporarily enhance flexible electrode rigidity by supporting the flexible material as close as possible to the probe tip^42,43^. To achieve this support during NHP insertion, we added a second “support tube” consisting of a 23 G needle (Fig. 2A). In the full system, the microwire shuttle, support tube, and guide tube fit together concentrically, creating a telescoping mechanism (Fig. 2A, B). In our design, the microwire threaded the flexible array and also formed the core of the telescope (Fig. 2A). Critically, the threaded Parylene-C probe runs along the outside of the support shuttle, pressed between the walls of the two steel tubes (Fig. 2A). Therefore, during microwire retraction, the Parylene-C was held in place by the guide tubes, enhancing rigidity and promoting de-threading of the tungsten shuttle while the flexible array remained free-floating in brain tissue (Fig. 2B, C).

Our full implantation assembly consisted of a commercial manipulator onto which we mounted a custom 3D-printed microdrive that housed the microelectrode array, headstage, and telescope components (Fig. 2A, Supplementary Fig. 3). This yielded a total of four operable axes for independent control of each component of the telescope, enabling fine insertion of our flexible arrays and stable removal of the shuttling mechanism. Benchtop testing of this multi-step process in agar is shown in Fig. 2B, C. First, we used the commercial manipulator to advance the entire system through a chronic recording chamber toward the brain until the guide tube penetrated dura. Next, we used the microdrive to deliver the threaded flexible array, microwire, and support tube through the guide tube and into the brain as one unit. At this point, we retracted the tungsten microwire with a second drive block built into the adapter, leaving the flexible array and smaller support tube in the brain. Following microwire retraction, we then retracted the support tube into the larger guide tube, leaving only the flexible array in tissue at the desired recording depth (Fig. 2B, C). In 29 in vivo attempts in two animals, we successfully penetrated to the recording depth in 24 sessions (82.8%, Fig. 2D, Table 1). Retraction was also highly reliable. Our flexible arrays remained in the brain in 87.5% of successful penetrations (21 sessions total, Fig. 2D, Table 1). In all successful retractions, we also recorded single-unit activity from the free-floating array (Fig. 2D). Retractions that failed (*n* = 3) were due to the tungsten wire tearing the thread-hole of the flexible array, most likely during the advancement into tissue (Fig. 2). If any stage of implantation failed (Fig. 2D), the nonsurgical setting allowed us to thread a new probe and attempt another insertion on the same or a subsequent day. These results demonstrate that our telescopic insertion process enables reliable delivery into large-brained animals through intact dura and without the need for a new craniotomy.

**Table 1:** Summary of implantation outcomes

Animal ID	Brain Area	Total Attempts (N)	Successful Penetrations (N, %)	Successful Retractions (N, % of Penetrations)	Behavioral Recordings (N, % of Retractions)
**Animal R**	PFC	9	7 (77.8%)	6 (85.7%)	5 (83.3%)
**Animal R**	PPC	12	10 (83.3%)	9 (90%)	8 (88.9%)
**Animal O**	PFC	8	7 (87.5%)	6 (85.7%)	5 (83.3%)
**Overall Total**		29	24/29 (82.8%)	21/24 (87.5%)	18/21 (85.7%)

### Single-unit recordings using flexible arrays in awake macaques

**Fig. 3:**
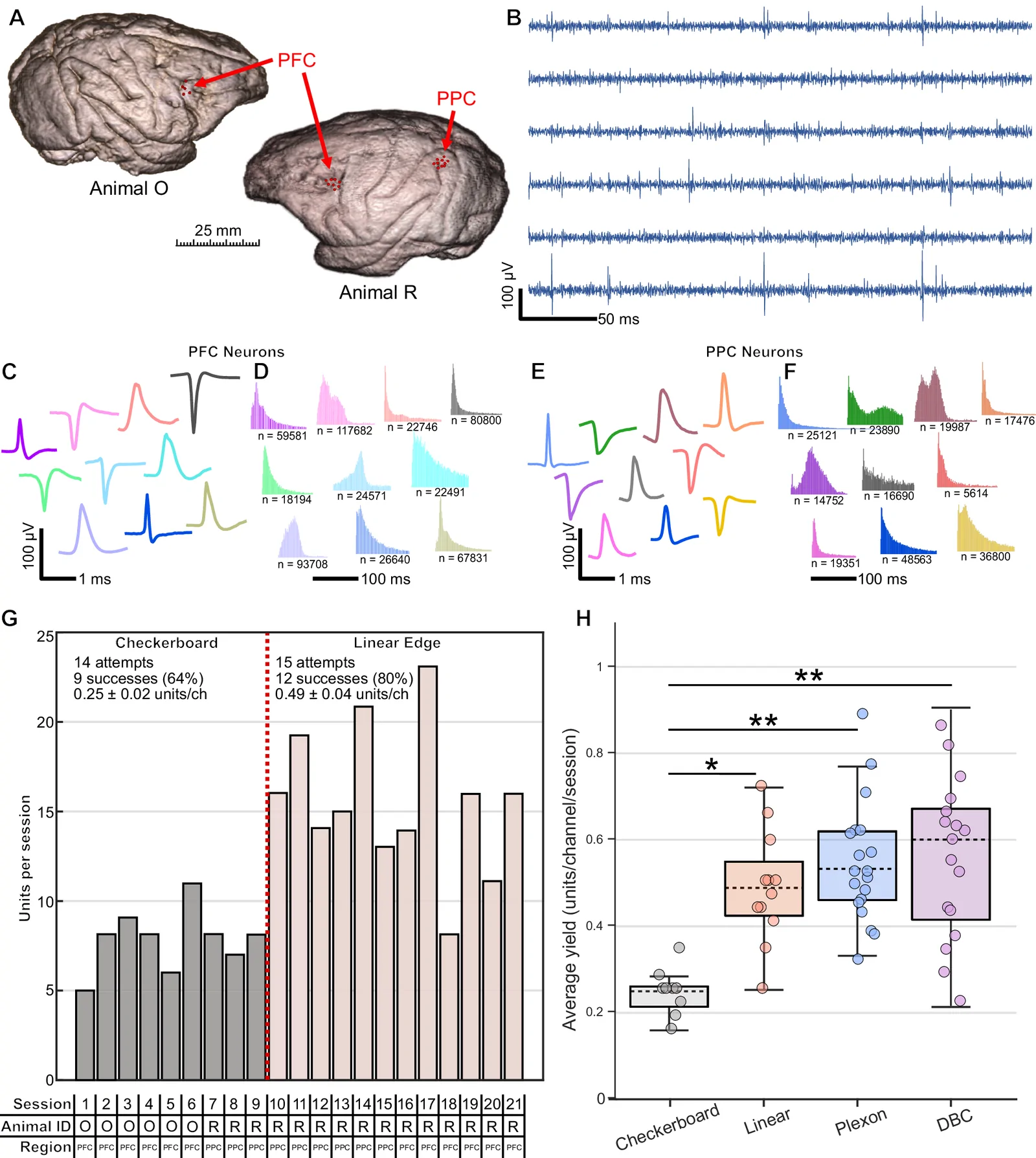
Flexible microelectrodes record single-unit activity across cortical regions. **A** MRI brain scan for both animals overlaid with the locations of successful recordings with flexible microelectrodes. **B** Representative spike band data from a select set of electrodes in the PFC of animal O. **C**,** D** Representative waveforms and inter-spike intervals detected in the PFC of animal O. **E**,** F** Representative waveforms and inter-spike intervals detected in the PPC of animal R. **G** Number of single units detected in each recording session. Sessions are organized chronologically. The red line denotes the switch from the checkerboard design to the linear-edge design. The bottom table denotes the session number, animal ID, and brain area. Recording attempts, successes, and average units per recording channel per session are also noted. **H** Box plots summarize recording-site yield for each probe type (checkerboard flexible, *n* = 9 sessions; linear flexible, *n* = 12 sessions; Plexon, *n* = 19 sessions; DBC, *n* = 17 sessions). Each point represents one independent recording session. The center line indicates the median, the box bounds indicate the 25th and 75th percentiles, whiskers extend to the most extreme data points within 1.5× the interquartile range, and points beyond the whiskers are plotted individually as outliers. Average recording-site yield differed significantly across probe types (one-way ANOVA, F(3,57) = 6.14, two-sided, *p* = 0.0011). Linear flexible probes yielded 0.49 ± 0.04 units/channel/session, compared with 0.25 ± 0.02 units/channel/session for checkerboard probes, 0.55 ± 0.03 units/channel/session for Plexon probes, and 0.60 ± 0.06 units/channel/session for DBC probes (mean ± SEM). Post hoc Bonferroni-corrected comparisons showed that checkerboard probes yielded significantly fewer units/channel/session than linear flexible probes (*p* = 0.017), Plexon probes (*p* = 0.0014), and DBC probes (*p* = 0.0018), whereas linear flexible probes did not differ significantly from either Plexon or DBC probes (both *p* = 1.0).

Having established reliable implantation procedures, we next assessed the quality and yield of single-unit activity. Our insertion process forgoes the need for new surgical interventions and therefore allowed us to perform repeated recordings in *n* = 2 awake, head-fixed macaques performing an oculomotor delayed response (ODR) task. We performed in vivo recordings across two brain regions, prefrontal cortex (PFC) and posterior parietal cortex (PPC), in three different recording chambers (PFC in animals R and O, PPC in animal R, Fig. 3A). Following retraction of the tungsten shuttle, we stably recorded multiple neurons that exhibited robust spiking (Fig. 3B). In both brain areas, extracted waveforms and inter-spike intervals displayed single-unit features (Fig. 3C–F). Across all 21 successful recording sessions, we isolated 232 single units; 18 of these sessions also contained usable behavioral data (Table 2). We observed units distributed across the length of the flexible array with an amplitude and signal-to-noise ratio (SNR) comparable to previously reported flexible microelectrodes (Supplementary Figs. 5–7). These results indicate that the insertion process preserved the ability to record well-isolated single-unit activity throughout the targeted recording region.

**Table 2:** Summary of successful flexible-array recording sessions and isolated single units

Animal ID	Sessions	PFC Neurons	PPC Neurons	Total
O	6	42	n/a	42
R	15	77	113	190
Total	21	119	113	232

The repeatability of our implantation approach allowed us to troubleshoot different electrode array geometries for optimizing single-unit yield (Fig. 3G, H). We began with a three-column checkerboard design that emphasized electrode density for spike detection across multiple sites simultaneously. Surprisingly, we found that this geometry resulted in a low spike yield (Fig. 3G, H). We hypothesized this could be due to the electrode site distance from the probe edge, which has been observed with silicon arrays^44,45^. We then redesigned and fabricated a new batch of flexible arrays with a linear-edge design, where we placed all metal contacts only 3 µm from the Parylene-C edge (Fig. 1). This geometric change immediately doubled the single-unit yield per recording site (0.25 ± 0.02 units/ch for the checkerboard design, 0.49 ± 0.04 units/ch for the linear design, mean ± SEM) and significantly increased the number of units recorded per session (Fig. 3G, H). This increase was unlikely to result solely from the tapered geometry and reduced width of the linear-edge design near the probe tip. Although the linear-edge design tapers from 275 to 50 μm, recording site yield did not vary significantly with depth (Supplementary Fig. 4, *p* = 0.886 linear, *p* = 0.195 checkerboard). Instead, the linear-edge design yielded more units than the checkerboard design in both the shallowest and deepest thirds of the array (Supplementary Fig. 4).The increased yield cannot be explained by impedance differences between the two designs. Both array designs exhibited a statistically identical 1 kHz electrochemical impedance (Supplementary Fig. 2) and we observed no clear relationship between the recording site impedance and median channel SNR (ρ = 0.144, *p* = 0.152) or spike amplitude (ρ = −0.105, *p* = 0.289) (Supplementary Fig. 7, *n* = 6 sessions, 105 recording sites). This provides substantial evidence that the design of flexible microelectrode arrays can influence recording quality.

Furthermore, we compared our single-unit yield results for flexible arrays to commercial rigid electrode arrays. In addition to the 21 recordings performed with flexible arrays in animals O and R, we performed 35 additional acute recordings in the same animals and chambers using a combination of 32-channel Plexon S/V-Probes and 128-channel DBC Deep Array Probes. Both rigid probes are fabricated from stainless steel with a 200-μm shank diameter and have an electrode spacing similar to our flexible devices. Across probe types, average recording-site yield differed significantly (one-way ANOVA, *F*(3,57) = 6.14, *p* = 0.0011; Fig. 3H). Bonferroni-corrected post hoc comparisons showed that the checkerboard probes yielded fewer units per channel per session than linear flexible probes (*p* = 0.017), Plexon probes (*p* = 0.0014), and DBC probes (*p* = 0.0018). Linear flexible probes did not differ significantly from Plexon or DBC probes (both *p* = 1.0).

Overall, our recording success rate also improved throughout the study as we refined the implantation approach. Out of 15 total attempts using the linear design, we recorded single-unit activity in 12 sessions, for a success rate of 80% compared to 64% for the checkerboard experiments (Fig. 3H). Together, telescopic insertion and the optimized flexible-electrode design produced high-quality recordings suitable for studying neural activity in behaving primates.

### Flexible arrays exhibit multi-fold reductions in recording drift

**Fig. 4:**
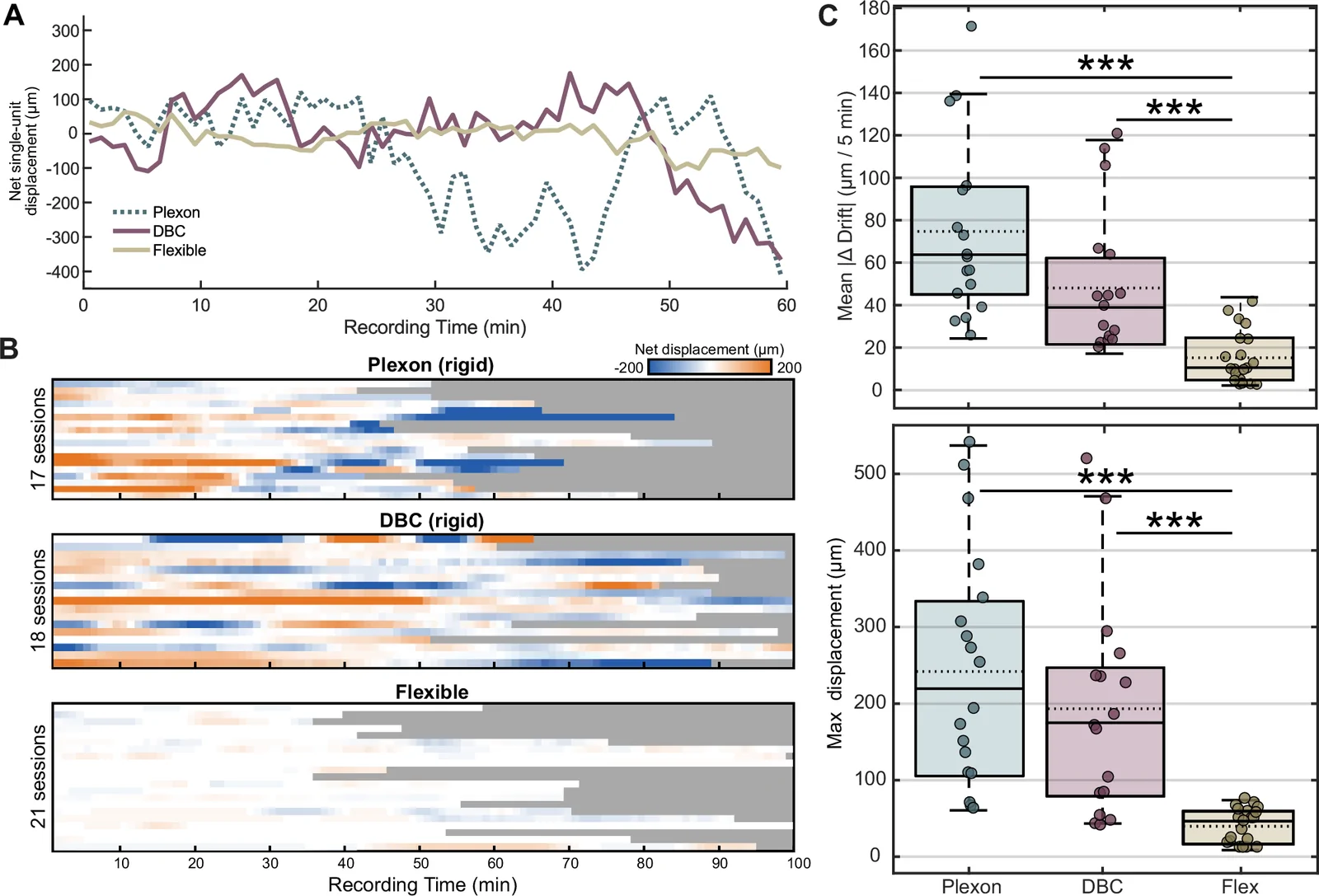
Flexible arrays have low recording drift compared to rigid electrodes. **A** Representative probe drift traces of three recordings for each probe type from animal R PPC chamber using a Plexon V-probe, Diagnostic Biochips 128-channel Deep Array probe, and our flexible array. **B** Heatmap showing median-centered population spike-depth traces over time for all independent recording sessions obtained with Plexon (*n* = 18 sessions), DBC (*n* = 17 sessions), and flexible arrays (*n* = 21 sessions) across both animals (O and R). Each row represents one independent recording session. Drift values are binned into 1-min windows. **C (Top)** Box plot summarizing the mean absolute five-minute change in the median-centered population spike-depth trace for each independent recording session by probe type (Flexible, *n* = 21 sessions; Plexon, *n* = 18 sessions; DBC, *n* = 17 sessions). Each point represents one independent recording session. The center line indicates the median, the box bounds indicate the 25th and 75th percentiles, whiskers extend to the most extreme data points within 1.5× the interquartile range (IQR), and points beyond the whiskers are plotted individually as outliers. Across all four probe types, including the Neuropixels control dataset, five-minute drift differed significantly (two-sided Kruskal–Wallis test, χ²(3) = 35.14, *p* = 1.14 × 10⁻⁷). The main figure displays the within-animal flexible, Plexon, and DBC datasets; Neuropixels results are shown in Supplementary Fig. 8. Flexible arrays exhibited significantly lower five-minute drift than Plexon probes (two-sided Wilcoxon rank-sum test with Bonferroni correction, *p* = 3.10 × 10⁻⁶) and DBC probes (*p* = 1.89 × 10⁻⁴). **(Bottom)** Box plot summarizing maximum displacement, defined as the peak-to-trough range of the median-centered population spike-depth trace for each independent recording session (Flexible, *n* = 21 sessions; Plexon, *n* = 18 sessions; DBC, *n* = 17 sessions). Each point represents one independent recording session. Box plot elements are defined as above. Maximum displacement differed significantly across probe types (two-sided Kruskal–Wallis test, χ²(3) = 36.06, *p* = 7.27 × 10⁻⁸). Flexible arrays exhibited significantly lower maximum displacement than Plexon probes (two-sided Wilcoxon rank-sum test with Bonferroni correction, *p* = 1.59 × 10⁻⁶) and DBC probes (*p* = 1.57 × 10⁻⁵).

While flexible electrodes are theorized to move with brain micromotions and reduce drift, direct quantitative comparisons with rigid probes have not been performed. Our non-surgical approach uniquely enabled us to perform this comparison by recording from the same craniotomies with both flexible and rigid electrode types. For acute recordings from our flexible electrodes, Plexon S/V probes, and DBC Deep Array probes, we quantified probe-relative recording drift using a population spike-depth metric derived from Kilosort template depths (Fig. 4A, “Methods”). Probe-relative population drift traces for all rigid and flexible recording sessions is shown in Fig. 4B. A dramatic reduction in drift for flexible arrays is immediately apparent. We further quantified this data by calculating the mean absolute drift across 5 min bins and the maximum peak-to-trough displacement for each recording session (Fig. 4C). Both metrics indicate a multi-fold reduction in drift for flexible microelectrodes (maximum displacement: 219.4, 175.0, 39.45 µm; 5-min drift magnitude: 63.7, 38.9, 7.45 µm/5 min for Plexon, DBC, and flexible probes, respectively, *p* < 0.001; Fig. 4C). Therefore, flexible arrays exhibited significantly lower drift than both rigid probe types across both metrics.

To rule out the possibility that the reduction in probe-relative spike-depth drift was driven primarily by probe size rather than mechanical compliance, we performed additional recordings using Neuropixels probes, which have a substantially smaller form factor (70 µm shank width, 20 µm thickness) than the stainless-steel Plexon and DBC probes (approximately 200 µm diameter). These recordings were obtained in two separate animals from the flexible, Plexon, and DBC datasets and should therefore be interpreted cautiously. Nevertheless, across animals, Neuropixels recordings exhibited drift metrics comparable to those observed with the rigid steel probes (Supplementary Fig. 8, Table S1). In contrast, Neuropixels drift remained substantially greater than that observed with flexible arrays (maximum displacement, *p* = 0.00002; 5-min drift magnitude, *p* = 0.00007, Table S1). To determine whether the reduced drift observed with flexible probes was consistent across animals, we separated the drift calculation by subject. Flexible probe recordings consistently showed lower drift compared to rigid probes, while no significant differences in drift metrics were observed between animals (Supplementary Fig. 8). Together, these findings are consistent with the interpretation that mechanical compliance with surrounding brain micromotion contributes to the reduced probe-relative drift observed with flexible arrays.

### Flexible arrays capture task-modulated single-unit activity during working memory

**Fig. 5:**
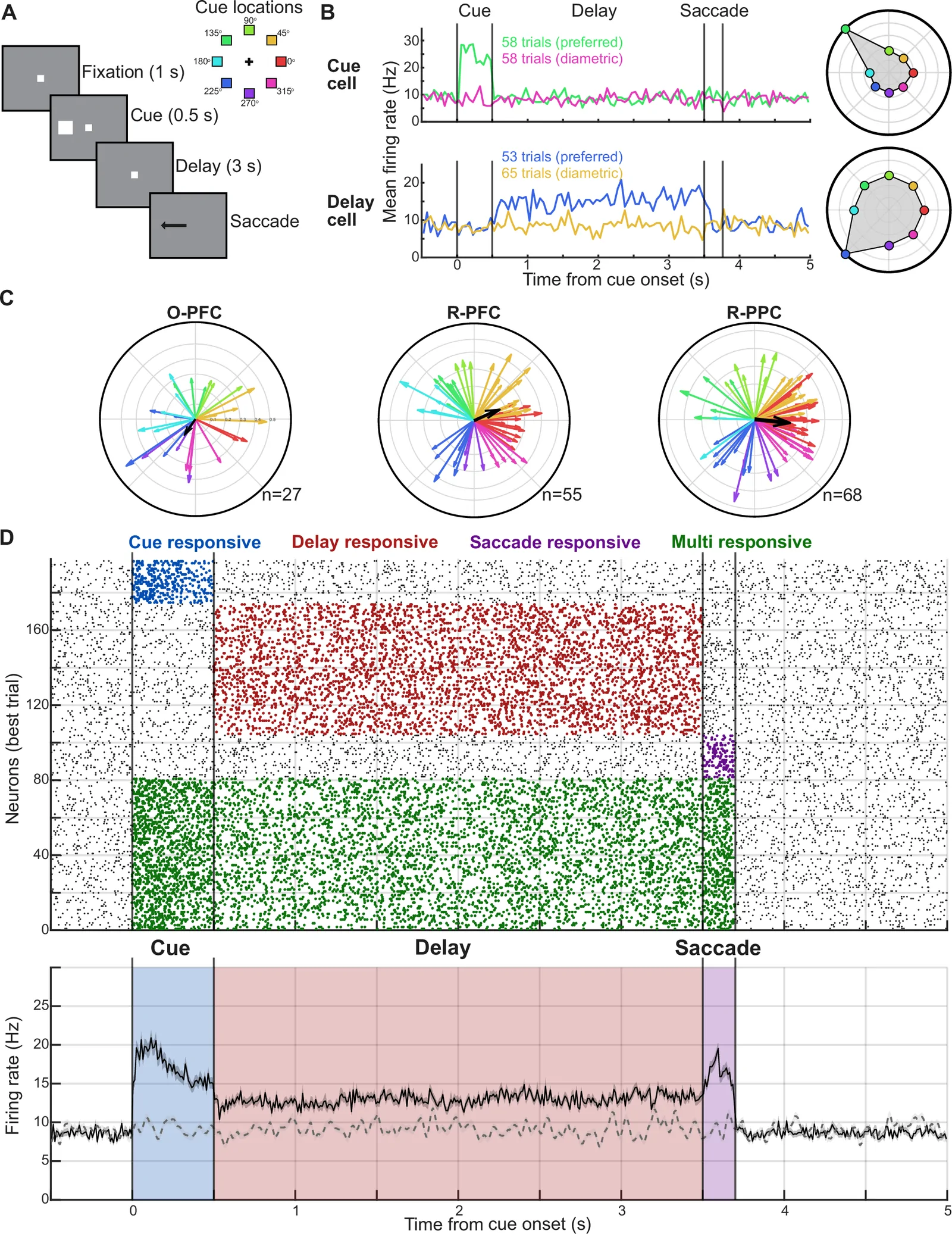
Task-relevant activity recorded from flexible array. **A** Left: Sequence of events in the ODR task. The animal is required to maintain fixation for 1 s to observe the cue stimulus, remain fixated for the delay period of 3 s, and make a saccade to the remembered cue location after the delay period, indicated by the disappearance of the fixation point. The animals were rewarded for the successful completion of the trial. Right: Cue stimuli locations are shown in a ring with different colors. **B** Representative cue-response (top) and delay-responsive (bottom) cells. Plots show the peristimulus time histogram (PSTH) for trials with the cue in the preferred location compared to the target 180° away in the diametric location. Compass plots show cell tuning. **C** Compass plot showing tuning of all single units selective for the delay period recorded in each brain area. Each arrow’s direction denotes the angle of tuning and length indicates the strength of tuning for each unit. Each arrow color indicates the spatial selectivity of that neuron. **D** Top: Raster plot showing single-trial activity of all single units that showed behaviorally relevant firing, organized as cue, delay, saccade, and multiple epoch responsive units. Each row represents one trial from one neuron, with rows grouped by the neuron’s task-response category and aligned to cue onset. Bottom: PSTH for the trials shown in the raster above, across all single units. Solid trace shows the average activity for all units’ preferred cue location. The dashed line shows average activity for all units for the diametric location.

To verify that our flexible arrays capture task-relevant neural activity, we analyzed single-unit responses during the ODR task (Fig. 5A). The ODR task is a well-established approach for probing working memory, which is a significant function of PFC and PPC^46,47^. From 21 neural recording sessions, 18 also contained sufficient behavioral data during execution of this task (Fig. 2D). In the three failed sessions, recordings were obtained, but due to hardware malfunctions we were unable to correlate electrophysiology with behavior (Fig. 2D, *n* = 1 behavior software failure during behavior, *n* = 2 eye tracking hardware failure). We classified single units into four commonly identified subgroups: cue responsive, delay responsive, saccade responsive, or responsive to multiple task epochs within the same trial, based on their firing rate (see Methods). Furthermore, we identified neurons showing tuning to location during the delay epoch, as is characteristic of PFC neurons^48^.

From 232 total single units recorded across sessions in PFC and PPC, 197 were identified as task-responsive (see Methods). These included 23 cue-responsive, 70 delay-responsive, 23 saccade-responsive, and 81 multi-epoch neurons. We quantified the spatial tuning of these cells during the delay period and found strong single-neuron tuning distributed across target locations (Fig. 5B, C), with a slight bias in population tuning toward the contralateral field-of-view (Fig. 5C). The distribution of functional cell types did not differ between PFC and PPC (χ²(4) = 1.12, *p* = 0.89). We compiled a population-averaged firing rate for all task-responsive cells (Fig. 5D). Individual neurons maintained consistent firing patterns across the recording session (Supplementary Figs. 9-10). These results confirm that our flexible arrays reliably capture task-modulated neural activity with response properties characteristic of PFC and PPC, demonstrating their suitability for studying cognitive neuroscience in behaving primates.

## Discussion

We successfully developed a flexible electrode array design suitable for acute NHP recordings and demonstrated a non-surgical, repeatable approach for inserting them in awake, behaving NHPs. We attribute our improved success over previous studies to our telescopic approach for implantation, which reliably delivered free-floating flexible arrays through intact dura. This insertion approach allowed us to optimize the electrode array design to maximize single-unit yield. In addition, we quantitatively showed that highly flexible electrode arrays have similar single-unit yields when compared with traditional rigid electrodes, but superior recording stability. Recording stability improves the fidelity of single-neuron firing rate measures, such as those we quantified in our study, but also measures of synchronous activity between neurons, such as spike-count correlation and cross-correlation^49^. Combined, our methods led to repeated acute recordings over several weeks across multiple brain regions in NHPs performing cognitive tasks and hundreds of neurons with task-modulated activity during the working-memory task.

Our work substantially lowers barriers to flexible electrode adoption in the broader NHP neurophysiology community. Our non-surgical, acute approach provides an accessible entry point for laboratories to adopt flexible electrode technology without high-risk surgeries. Our approach works through standard recording chambers with intact dura, allowing researchers to perform flexible electrode recordings using the same infrastructure and workflows they already use for rigid probes. We used a combination of a commercial micromanipulator, off-the-shelf parts, and a 3D printed microdrive with designs that we have made freely available (“Methods”). This compatibility means laboratories can integrate flexible arrays without major equipment investments, specialized surgical training, or procedural overhauls. In addition, this also means that animals with recording chambers that are not currently being used for active studies can be repurposed for flexible electrode testing. In addition to the practicality of our approach, previous flexible electrode demonstrations in NHPs have not reported the outcomes of all implantation attempts, suggesting that recordings were not only high-risk but potentially unreliable. Our study reports the outcomes of all implantation attempts, including unsuccessful penetrations and retractions (Fig. 2D). From 29 total attempts in awake animals, we achieved single-unit recordings in 21 sessions (72% success). Notably, after optimizing the probe design, we successfully implanted the array and recorded single-unit activity in 12 of 15 attempts (80% success, Fig. 3G), and we expect this yield to increase as we continue to minimize human error (see Supplementary Discussion). These data provide NHP groups clear expectations for success rates and failure modes, enabling realistic planning and iterative improvement rather than reliance on selectively reported successes. Finally, we performed side-by-side recording comparisons with traditional rigid microelectrode arrays. Our results demonstrate that free-floating flexible arrays have similar single-unit yields per recording site, but superior stability of single-unit recordings, likely because these tissue-like interfaces move with natural brain micromotion. Combined, our work provides the technical innovation to overcome implantation challenges and the quantitative evidence to validate theoretical advantages, further establishing flexible microelectrode arrays as a practical, accessible technology for NHP neuroscience.

Building on the acute recording and insertion methods developed here, a key area of future development is a platform for chronic, large-scale, and highly stable longitudinal recordings. While the flexible arrays we used in this study have only 32 recording sites, the microfabrication process is scalable to larger channel counts. However, our results suggest that scaling comes with important design considerations, such as metal contact placement and overall shank width. The width can be partially mitigated using a multi-layer fabrication approach as shown in other studies^50–54^. Importantly, increasing the electrode count also non-trivially increases the complexity and size of the I/O interface, which should be kept small to easily anchor to the recording chamber. Commercial small-form-factor 128-channel headstages, including the Plexon HST/128DS Gen 1 and the Cambridge Neurotech Mini-Amp_128, could support this scaling. With these considerations, it is highly feasible to scale to a 128-channel shank that is compatible with our implantation approach and with a form factor suitable for regular use in academic NHP laboratories.

A crucial next step in this work is to adapt our insertion and recording approaches for chronic implantation. The non-surgical insertion approach described here has unique advantages compared to traditional methods. For example, rather than risking a new craniotomy, animals with existing craniotomies can be used for developing chronic insertion techniques and assessing chronic recording stability. In addition, techniques for anchoring electrodes in tissue can be tested for days or weeks at a time, followed by removal and re-implantation of a new array. However, the chronic integration of flexible microelectrodes in NHPs poses unique challenges that must be considered. For example, the long-term inflammatory response to implanted flexible arrays in large-brained animals remains poorly documented. In rodents, it is well established that thin, flexible electrodes implanted on the order of months integrate well with neural tissue, demonstrated by healthy neurons within microns of the array with minimal aggregation of microglia and reactive astrocytes^20–26,29,55,56^ Indeed, when implanted for more than 175 days in mice, our own flexible electrodes also show minimal neuronal displacement and astrocyte aggregation near the implantation site (Supplementary Fig. 11). Unfortunately, similar replications in NHPs with flexible electrodes, while promising, remain sparse and largely unquantified. Additional studies are required to assess whether flexible electrodes provide (i) biological stability, (ii) materials stability, and (iii) recording stability in months- and years-long contexts in large-brained animals. Our work with acute recordings here suggests that the recording stability offered by flexible electrodes, paired with the possibility of minimal immune response when chronically implanted, may yield excellent long-term recording performance in NHPs.

Overall, our study demonstrates that flexible electrodes can be reliably implanted with high yield in behaving NHPs and provide substantially improved acute stability relative to rigid microelectrodes. By lowering the technical barriers to reliable flexible-array recordings, our approach lays the groundwork for future studies that can follow how individual neurons and population-level computations evolve over learning, development, and cognitive change.

## Methods

### Flexible microelectrode fabrication

We developed a custom design for fabricating ten Parylene-C–based probes simultaneously on 4-inch silicon (Si) wafers using a standard photolithography process. We first cleaned each wafer by soaking it for 5 min in acetone, followed by 5 min in isopropyl alcohol (IPA). Afterward, we dried the wafers with nitrogen and then O₂-plasma cleaned them for 1 min (Trion–Phantom RIE). We deposited approximately 3 µm of Parylene-C using chemical vapor deposition (Specialty Coating Systems) with 3.5 g of dimer for four wafers at once. To improve surface adhesion and remove any residual monomers, we vacuum-baked the samples overnight at 160 °C at 30 mm Hg.

We used mask photolithography to pattern metal recording contacts, leads, connection pads, and the through-hole at the probe tip. We repeated the cleaning sequence (acetone, IPA, drying, and 10-s O₂-plasma treatment) to prepare the Parylene-C surface. A 450 nm layer of LOR7A lift-off resist (Kayaku) was spin-coated and baked at 160 °C for 10 min. Subsequently, a 1.3 µm layer of S1813 (Microposit) was spin-coated and baked at 115 °C for 1 min. Using a mask aligner (Karl Suss Ma-6), we exposed the photoresist with the metal pattern at 110 mJ/cm² and developed it for 1 min in MF-319 (Kayaku), then rinsed it in deionized (DI) water. We O₂-plasma cleaned the wafers for 10 s to improve metal adhesion, then deposited 200 nm of Pt at approximately 2.5 nm/min using DC sputtering (AJA ATC-2200). We lifted off the metal layer for 2 h at 80 °C then at room temperature overnight in Remover 1165 (Microposit), then rinsed in DI water. We O₂-plasma cleaned the surface again for 10 s to remove any solvent residue and reactivate the surface. We chose not to apply additional surface treatments to the Pt microelectrodes. Although this increased the variability of electrochemical impedance across recording sites (Fig. 1, Supplementary Fig. 2), impedance was not significantly correlated with channel SNR or spike amplitude (Supplementary Fig. 7).

We encapsulated the patterned metal layer by depositing another approximate 3 µm of Parylene-C using the same method, then vacuum-baked the wafers again overnight at 160 °C and 30 mm Hg. To define the recording sites, connection pads, and probe outline, we first deposited a photoresist etch mask onto the parylene-Pt-parylene stack. We began by repeating the cleaning steps, then spun 12 µm of SPR 7.0–220 (Kayaku) and prebaked the sample at 70 °C for 150 s, then at 115 °C for another 150 s. After cooling for 3 min, we exposed the resist twice at 280 mJ/cm²: once with a mask revealing the contacts and pads, and once for the probe outline. We let the sample rest for 35 min, post-baked it at 70 °C for 90 s, then at 115 °C for 90 s, and rested it again for 10 min. We developed the resist in MF-319 for 5 min.

We etched the Parylene-C using high-power O₂ plasma (250 W RIE, Trion Minilock) to expose the metal contacts (below the top 3 µm layer of parylene) and define the probe outline (through 6 µm of parylene) simultaneously. To prevent overheating, we alternated 2 min of etching with 1 min of cooling, repeating this cycle 10 times for a total etch time of 20 min, or until the Si wafer was visibly clear of Parylene-C. To avoid over-etching, we designed the recording-site etch mask with a 25 µm diameter, 5 µm smaller than the metal layer, which expanded to a final diameter of 30 µm during etching. Afterward, we stripped the remaining photoresist in acetone, rinsed in IPA, and dried the wafers with nitrogen.

To improve handling of the long, thin polymer devices, we added a photolithography step to thicken the probe backend. We activated the Parylene-C surface with a 30-s O₂-plasma clean, spun SU-8 2010 (Kayaku) to 12 µm thickness, and baked the wafers for 3 min at 95 °C. Using a mask aligner, we exposed the resist with 135 mJ/cm², baked for 4 min at 95 °C, developed in SU-8 developer (Kayaku) for 3 min, and rinsed in IPA. To remove microcracks, reduce stress, and improve adhesion to Parylene-C, we hard-baked the wafers in a stepped ramp: 2 min each at 100 °C, 110 °C, and 120 °C, followed by 1 h at 130 °C. This SU-8 layer reinforced the backend and surrounded the probe’s through-hole tip, while the implantable shank remained Parylene-C only, preserving flexibility.

### Probe packaging

We released the Parylene-C probes by submerging the wafer in DI water and peeling the devices using fine-tip surgical tweezers. To minimize damage to the conducting layer, we designed a small handle in the backend region that included only SU-8 and Parylene-C with no platinum, and we used this area to begin peeling. Once the handle separated from the wafer surface, the rest of the device released easily from the wafer. The SU-8 layer reduced curling compared to 6 µm-thick Parylene-C probes and made handling easier. In an effort to minimize curling further, we annealed the probes between Teflon sheets using a vacuum bake at 160 °C for 2 h, at a pressure of 30 mmHg^57^.

After drying, we bonded the probe backend to a laser-cut 0.010-inch-thick polyether-ether-ketone (PEEK) film (CS Hyde, 37-10F-24” × 24”) using superglue, and any excess Parylene-C was trimmed off the backend^58^. This PEEK support allowed stable insertion and clipping into a 32-channel zero-insertion-force (ZIF) connector (Hirose FH12-32S-0.5SH) mounted on a custom 1.0 × 0.5-inch printed circuit board (PCB). We soldered a ground pin and a 32-channel Omnetics connector (A79022-001) to the PCB, allowing it to interface with a 32-channel Intan amplifier (RHD 2132).

### Electrochemical impedance

For flexible microelectrodes, we tested benchtop impedance using three-electrode electrochemical impedance spectroscopy in PBS (Gamry Interface 1010E, 10 mV RMS amplitude, 1–10,000 Hz sweep). The recording site was used as the working electrode, an Ag/AgCl electrode as the reference electrode, and Pt wire as the counter electrode. For commercial rigid electrodes, the reported 1 kHz electrochemical impedances were recorded using on-chip measurements with the Intan RHD headstage and submerging the probe in saline with respect to an Ag/AgCl electrode.

### Experimental model and subject details

Two male rhesus monkeys (*Macaca mulatta*), approximately 9 years old and weighing 9–14 kg were used in this study for flexible, Plexon, and DBC electrode recordings (Animals O and R). Two additional animals (P and V) of similar age and sex were used for Neuropixels recordings. Animals were single housed in communal rooms, and they had sensory interactions with other monkeys. Mouse experiments were in adult Thy1-EGFP and Thy1-GCaMP6s animals (The Jackson Laboratory, #007788 and #024275, respectively). Mice were kept on a 12-h light/dark cycle in conventional housing with unrestricted access to food and water. All surgical and animal use procedures were reviewed and approved by the Vanderbilt University Institutional Animal Care and Use of Laboratory Animals and the National Research Council’s Guide for the Care and Use of Laboratory Animals.

### NHP surgery

Animals were implanted with a 20-mm-diameter recording cylinder during previous studies^59,60^, and we continued the use of these chambers for the current study. Both animals used for recordings in this study had a chamber over the dorsolateral prefrontal cortex (Animals R and O) and one subject had a posterior parietal cortex chamber (Animal R). We registered CT and MR images to localize the cylinder and electrode penetrations. We aligned anatomical T1 images to the high-resolution NIH Macaque Template (NMT) using AFNI @animal_warper^61,62^. We used 3D Slicer to find the projection of the recording cylinder on the brain surface, then localized electrode penetrations using the grid locations relative to the recording cylinder.

PFC for both animals targeted the dorsolateral region (Brodmann areas 8a and 46) along the bank of the principal sulcus using previously acquired MRI scans with chamber coordinates mapped to the brain. PPC recordings primarily targeted the intraparietal sulcus using the same registration with chamber coordinates.

### NHP flexible electrode implantation

Advancing our flexible microelectrodes into the brain required a custom “microdrive” adapter that we attached to a conventional micromanipulator (MO-97A, Narishige Corp.). We designed and 3D printed the microdrive similar to other instrument designs^63,64^. The designs are openly available online (https://github.com/GonzalesLabVU/NHPflex2025).

The Narishige microdrive consists of a dovetail interface between the two rails of our 3D printed adapter: a main driver that held a sharpened 19 G guide tube and a second driver that mounted our flexible probe, tungsten wire, support tube, PCB, and headstage. Each rail could be operated independently. We used the main driver to lower the entire assembly toward the brain for initial penetration of the dura using the guide tube. We followed this by using the second rail for fine-scale driving of the flexible probe threaded to a tungsten microwire (FHC Inc., UEWLGGSE4N1E) and encased by the 23 G blunt support tube. After advancing the probe tip to the desired recording depth, we used our third custom driver that retracted the tungsten microwire from the brain via a screw. The 23 G encasement facilitated detachment of the tungsten and Parylene-C. Once the tungsten microwire was removed, we retracted the 23 G encasement tube into the 19 G guide tube, leaving the flexible microelectrode freely in cortical tissue. The guide tube remained inserted in dura for the duration of the recordings.

Threading of the flexible array occurred prior to implantation. We first pre-routed the tungsten microwire through the microdrive and 23 G support tube, then threaded the microwire tip to the flexible array and temporarily bonded them using poly-ethylene glycol (PEG) (Sigma, 94646-250G-F). We aligned the microwire such that it protruded from the support tube by approximately 5 mm. During insertion, we estimate it takes approximately 5 min for the PEG to fully dissolve. During this period, recording signals are temporarily inhibited, making it challenging to record during the insertion process.

### Mouse surgical procedures and flexible electrode implantation

Mice were first implanted with a headpost. Mice were deeply anesthetized with 5% isoflurane, then transferred to a stereotax. During surgery, anesthesia delivered through the nose cone was maintained at approximately 1.5% isoflurane with an oxygen flow rate 140 mL/min. An infrared warming pad (Kent Scientific) was used to maintain the body temperature. Carprofen (0.6 mg kg^−1^) was injected subcutaneously, and lidocaine injected under the scalp. Eye ointment was applied, and the scalp shaved and sanitized before the incision. We cleaned the scalp and applied Vetbond (3 M) to improve adhesion to the exposed skull. We then used dental cement (Stoelting) to adhere a custom-designed, 3D printed headpost to the skull (Formlabs V4 clear resin). After curing, we covered the entire area with Kwik-Sil silicone (World Precision Instruments). After 3 days of surgical recovery, animals were given an additional 5 days of habituation to the headpost.

After habituation, we anesthetized animals as above, administered carprofen, and performed a second stereotactic surgical procedure. For a ground electrode, we drilled a burr hole in the contralateral hemisphere, inserted a 0.5 mm diameter Ag/AgCl wire between the skull and dura, and used dental acrylic to seal the ground in place. We then drilled a 3 mm diameter circular craniotomy in the implantation region and removed the bone flap with fine forceps. To improve insertion of the flexible electrodes, we also sliced the dura with a sharp 23 G syringe needle. Flexible microelectrodes were shuttled into tissue with a tungsten microwire (50 µm diameter, A-M Systems 715500). Electrodes were temporarily adhered to the microwire using a 10% solution of poly(ethylene glycol) (Sigma, 94646-250G-F), similar to previous studies^65–67^. The microelectrodes were inserted into the brain with a digital micromanipulator (Sutter, QUAD) to a depth of approximately 1 mm. We then flushed the area with saline and retracted the tungsten wire. The excess length of the flexible probe was flattened on the surface of the brain and covered by a 3 mm coverslip, which was glued in place. We then attached the I/O board to a support mounted on the back of the headpost and fixed the two together with cyanoacrylate. The animal then recovered in a warmed cage. With this approach, the probe could be maintained in tissue for months while the animal freely roamed its home cage.

### Mouse histology

Animals were transcardially perfused with 10 mL of phosphate-buffered saline solution (PBS) followed by 10 mL of cold 4% paraformaldehyde in PBS. Brains were obtained and placed in 4% paraformaldehyde in the 4 °C refrigerator overnight. They were then rinsed three times in PBS and transferred to a 30% sucrose PBS solution until they sank to the bottom of the tube. Once equilibrated, brains were sectioned at 60 μm on a freezing sliding microtome (Leica SM2010R) and processed for immunohistochemistry. Specifically, sections were incubated in blocking solution (5% Bovine Serum Albumin, 0.3% Triton X-100 in PBS) for 1 h on a rocker, followed by overnight incubation at 4 °C with primary antibodies (1:1000 rat anti-GFAP, Invitrogen 13-0300 clone 2.2B10, RRID:AB_2532994; 1:1000 Alexa647-conjugated rabbit anti-NeuN, Abcam ab190565 clone EPR12763, RRID: AB_2732785; diluted in blocking solution). The following day, sections were rinsed 3 times for 10 min in PBS and incubated for 2 h at room temperature with a secondary antibody (Alexa Fluor 594 AffiniPure Goat Anti-Rat IgG (H + L), Jackson ImmunoResearch 112-585-003, RRID: AB_2338372; 1:500) diluted in blocking solution. Sections were then rinsed three times for 10 min before mounting. Sections were mounted using Prolong Gold antifade with DAPI (Invitrogen) and visualized on an epifluorescence microscope (BZ-X 710, Keyence) using a ×10 objective.

### Neurophysiological recordings

We collected electrophysiological recordings using our custom flexible microelectrodes with an Intan RHD 2132 headstage. For rigid recordings, we used both Plexon S/V probes and DBC 128-channel Deep Array probes. The Plexon probes used Intan RHD 2132 headstages, while the DBC probes use an integrated headstage that utilizes Intan RHD amplifiers. As with the flexible arrays, all rigid electrodes were implanted using a conventional micromanipulator (MO-97A, Narishige Corp.) and advanced through a guide tube. All recordings used an Open Ephys data acquisition system (Open Ephys) sampled at 30 kHz.

### Behavioral task

The animals sat head-fixed in a primate chair while viewing a 1920 ×  1080, 60 Hz monitor (Samsung QMC32C) positioned 69 cm from their eyes under dim ambient light. An infrared eye tracker (ISCAN ETL-200; ISCAN, Inc.) monitored eye position at 500 Hz with at least 0.5° precision at the center point. We implemented visual stimulus control, eye monitoring, and synchronization with neural data in MATLAB (MathWorks) using the Psychophysics Toolbox^68^. Animals were previously trained on an oculomotor delayed response (ODR) task^69^. Each trial began with fixation on a 0.1° white square at the screen center. We required fixation within a 3° window around the central point while presenting peripheral visual stimuli at radial locations. If fixation broke at any time, we terminated the trial and withheld reward. For the cues, a 1° white square appeared for 0.5 s at one of eight locations spaced by 45° around a 10° eccentric circle. The animals had to remember the cue location during a 3 s delay period. When the fixation point disappeared, they had 0.6 s to make a saccade to the remembered location and then hold fixation for 0.1 s within a 6° radius window centered on the cue. Correct trials earned a water reward. Animals typically performed 400–500 trials over a 1-h recording session, with approximately 90% correct performance.

### Ethics statement

All procedures involving nonhuman primates and mice were reviewed and approved by the Vanderbilt University Institutional Animal Care and Use Committee and were conducted in accordance with the National Research Council’s Guide for the Care and Use of Laboratory Animals.

### Quantification and statistical analysis

#### Neural data processing

All neurophysiological analysis and quantification were performed using MATLAB. These sets of analyses use the same methods as our previous studies on working memory^7,59,70–73^. All data were initially band-pass filtered from 500 to 8000 Hz. We identified spike waveforms and sorted single units using the automated spike-sorting software Kilosort 4^74^ (https://github.com/MouseLand/Kilosort) and manually curated the clustered data using Phy2 (https://github.com/cortex-lab/phy/).

#### Spike signal-to-noise ratio (SNR) analysis

Spike signal-to-noise ratio (SNR, Supplementary Fig. 5, Supplementary Fig. 7) was quantified based on the ratio of spike amplitude to background noise^75^. For each unit, SNR was defined as:

1 $${{SNR}}_{{spike}}=\frac{{{median}}_{k}\left(\left|\min \left({v}_{{AP}}^{\left(k\right)}\right)\right|\right)}{{\sigma }_{{AP}}}$$

Where:

2 $${\sigma }_{{AP}}={MAD}({v}_{{AP}})/0.6745$$

And:

3 $${MAD}\left({v}_{{AP}}\right)={media}{n}_{t}\left(\left|{v}_{{AP}}\left(t\right)-{media}{n}_{{t}^{{\prime} }}\left({v}_{{AP}}\left({t}^{{\prime} }\right)\right)\right|\right)$$

${v}_{{AP}}^{\left(k\right)}$ is the band-pass filtered (500–8000 Hz) action-potential waveform for spike k, aligned to the spike time. The median across spikes was used to provide robustness to outliers. ${\sigma }_{{AP}}$ is the background noise and estimated from randomly selected, spike-free segments of the recordings. MAD denotes the median absolute deviation computed from background activity.

#### Probe drift calculation

Drift (Fig. 4) was quantified directly from sorted spike data exported from Kilosort 4 for each recording session using each probe type (our flexible arrays, Plexon S/V-probes, Diagnostic Biochips Deep Array 128 channel probe, and Neuropixels). These values have been reliably used for drift comparisons in several previous studies^8^. The analysis was performed using custom scripts in MATLAB (MathWorks). For each probe during a recording, a population spike-depth trace was constructed by calculating the median template-assigned spike depth within consecutive temporal bins^76,77^, expressed in µm. For each template k, template depth was estimated from the spatial distribution of waveform energy across recording channels, defined as the signal-energy-weighted center of mass:

4 $${D}_{k}=\frac{{\sum}_{c}({E}_{k,c}{y}_{c})}{{\sum}_{c}{E}_{k,c}}$$

Where ${E}_{k,c}$ is the signal energy of template k on channel c, and ${y}_{c}$ is the channel’s vertical position. Each spike i was then assigned a depth ${d}_{i}={D}_{{k}_{i}}$ according to its template identity. Spike times were divided into consecutive temporal bins of 60 s, and for each bin b containing ${N}_{b}$ spikes (bins were included only if they contained at least 25 spikes), the median spike depth was calculated as:

5 $${M}_{b}={median}\{{D}_{i}:i\in {B}_{b}\},$$

Where ${B}_{b}$ denotes the set of spikes occurring within bin b. This median-centered population spike-depth trace was used as a measure of probe-relative recording drift over time. Maximum displacement for each recording session was defined as the peak-to-trough range of the median-centered population spike-depth trace:

6 $${D}_{{net}}={\max }_{b}\left({M}_{b}\right)-{\min }_{b}({M}_{b}),$$

This metric quantifies overall amplitude of the probe-relative movement ${D}_{{net}}$. To quantify the temporal change of probe motion, we computed the mean absolute change over 5-min intervals Using the same median-centered spike depth trace described above. Specifically, the median trace was sampled every five consecutive 1-min bins yielding a downsampled sequence ${M}_{5}(j)$. The 5-min drift magnitude was computed for each recording as:

7 $${\Delta }_{{drift}}={mean}(\left|{M}_{5}\left(j+1\right)-{M}_{5}\left(j\right)\right|)$$

To visualize probe drift dynamics across all recording sessions, a heatmap representation of median-center spike depth drift was constructed as follows. For each session, the drift trace was summarized by 1-min non-overlapping bins. Within each bin the median drift value across all samples within was computed. All sessions were capped at 100 min for session wise drift analysis. The box plots summarizing distribution of 5-min drift magnitude and peak-to-trough median-centered spike drift trace per session were aggregated, drawing interquartile range and whiskers extending to 1.5× the interquartile range.

#### Spike amplitude and single-unit yield

For each unit, we used the spike times from Kilosort 4 to extract a 1 ms window around each detected spike. We bandpass filtered these segments 500–8000 Hz and common average referenced to reduce any correlated noise. Spike amplitude was calculated as peak to trough voltage displacement during the window for each unit’s spike. Unit yield was quantified at both the channel and session levels. Channel-level yield was defined as the number of units assigned to each recording site. Session yield was defined as the total number of units detected during a recording session.

#### Neural firing analysis

We generated single-trial peristimulus time histograms (PSTHs) for visualizations by calculating spiking events of single units in 50 ms bins. We defined neurons as selective during any task epoch if they showed significantly different responses to the spatial location of the stimulus via a one-way ANOVA test on the firing rates across trials during that specific task epoch. In order to avoid false positives with sparsely spiking cells, we required that a selective neuron exhibit a firing rate of at least 4 spikes per second for its best stimulus location during the task period where the ANOVA test indicated a significant main effect. To identify each neuron’s preferred location, we computed the circular mean of the cue angles weighted by the neuron’s mean spike count during the delay period^78^. For each neuron, we computed:

8 $$T=\frac{{\sum }_{j=1}^{8}{{n}_{{j}^{e}}}^{{i\theta }_{j}}}{{\sum }_{j=1}^{8}{n}_{j}}$$

Where ${n}_{j}$ is the mean spike count during the delay period in response to the cue ${\theta }_{j}(\,\,j=1\ldots 8)$ and we extracted its modulus *T* and angle ${\theta }_{{pref}}:T=T{e}^{i{\theta }_{{pref}}}$. The angle ${\theta }_{{pref}}$ is our estimate of the neuron’s preferred location during the delay period.

#### Population firing rate

To illustrate how the population firing rate evolved over time (Fig. 5), we averaged the activity of all neurons recorded from all sessions for each neuron’s preferred cue location (the preferred cue for each neuron was chosen as mentioned in the above section). In other words, we aligned each neuron’s activity across all the trials for the preferred location which yielded the mean population firing rate^79^.

#### Statistical analysis

All statistical testing was performed in MATLAB. For boxplots, the upper and lower edges of the box represent the upper and lower quartiles of the data, respectively, and the line within the box represents the median. Whiskers extend to the most extreme observations within 1.5 × the interquartile range (IQR). Data distributions were assessed before hypothesis testing. Parametric tests were used when their assumptions were judged appropriate; otherwise, non-parametric tests were used. Two-group comparisons were performed using two-sample Welch’s *t* tests or Wilcoxon rank-sum tests, as appropriate. Comparisons involving more than two groups were performed using one-way ANOVA followed by Bonferroni-corrected post hoc tests, or Kruskal-Wallis tests followed by corrected pairwise Wilcoxon rank-sum tests, as appropriate. Effect sizes for parametric pairwise comparisons were quantified using Cohen’s *d*. Unless otherwise indicated, summary values are reported as mean ± SEM. Exact sample sizes, statistical tests, and *p* values are reported in the corresponding text or figure legends.

Recording sessions were treated as session-level observations. Because multiple sessions were obtained from the same animal, these observations were not fully independent at the subject level. Animal-separated analyses are provided in Supplementary Fig. 8 to assess consistency across subjects. No statistical method was used to predetermine sample size. Sample sizes were based on the number of successful recording sessions obtained during iterative development of the insertion and electrode designs and are reported for each analysis. Recording sessions were not randomized because the study included sequential engineering optimization of electrode geometry and insertion procedures. Investigators were not blinded to probe type during implantation, recording, spike sorting, or analysis.

### Reporting summary

Further information on research design is available in the Nature Portfolio Reporting Summary linked to this article.

## Supplementary information

Supplementary Information

Reporting Summary

Transparent Peer Review file

## Source data

Source Data

## Data Availability

All data supporting the findings of this study are available within the article and its supplementary files. Any additional requests for information can be directed to, and will be fulfilled by, the corresponding authors. Source data are provided with this paper.

## Code availability

The custom code and data used in this study can be found in the following GitHub repository: https://github.com/GonzalesLabVU/NHPflex2026. The code is publicly available under the MIT License.
