# Supplementary material for: Repeatable, low-drift recordings in behaving nonhuman primates using flexible microelectrodes
Source: Nat Commun. 2026 Sep 9;17:10638. doi: 10.1038/s41467-026-77421-3 (PMC13649665; doi:10.1038/s41467-026-77421-3)
Supplement: Supplementary file 1 [file 41467_2026_77421_MOESM1_ESM.pdf]

SUPPLEMENTARY MATERIAL

Supplementary material for:

Repeatable, low-drift recordings in behaving nonhuman primates using flexible microelectrodes

Woods, et al. 2026

Corresponding author:

Daniel L. Gonzales, daniel.gonzales@vanderbilt.edu

Document includes:

Supplementary Discussion

Supplementary Table 1

Supplementary Figures S1-S11

SUPPLEMENTARY DISCUSSION

Unreported studies with flexible arrays

Neuralink has publicly described large-scale implantation of flexible electrode arrays in nonhuman primates and humans. However, detailed peer-reviewed descriptions of the NHP implantation procedures, recording outcomes, and failure rates are limited. The present study instead focuses on a chamber-compatible approach that can be implemented using openly described components and workflows in academic neuroscience laboratories.

Flexible probe failure modes

The most prominent failure mode was flexible probe breakages. We report 3 probe breakages during the retraction phase of recordings. These failures occurred when inconsistent probe positioning along the microwire—specifically, when the Parylene-C was threaded too far up or seated unevenly—created mechanical stress points during shuttle withdrawal. The exact positioning of the probe relative to the microwire appears critical: too far up and the probe experiences excessive tension during retraction; too shallow and the probe may not remain securely attached during insertion. Other groups have addressed this challenge in rodents [1] and NHPs [2,3] by employing microwire geometries resembling a "thumbtack," where an enlarged head at a fixed position provides a consistent, defined attachment point that securely holds the flexible array tip in place. This design eliminates the variability in probe placement that we encountered and may further improve the implantation yield. However, the thumbtack alone is unlikely to provide reliable retraction and free-floating polymer arrays, as demonstrated in previous NHP studies [3].

Prior to implantation, approximately 10% of flexible probes also broke during preparation, specifically while manually threading probes under a microscope. This process is currently laborious compared to traditional rigid microelectrodes and future work should focus on automating or simplifying the threading process, perhaps through custom jigs or pre-assembled probe-shuttle combinations. It should also be noted that some groups have had success implanting in rodents without the need for threading and only using a temporary PEG adhesive between the polymer and microwire [4]. In unpublished work, our group performs implantations in this manner in mice as well; however, we have found threading to be critical for successful NHP implantations.

Electrode array design

Our investigation shows the first quantitative evidence that metal contact position along a thin polymer array affects recording quality, specifically single-unit yield. We made a data-driven decision to transition from our original checkerboard array design to a linear design based on both probe yield and electrode performance (Fig. S2, Fig. S4). Evidence of these geometric constraints has also been seen for silicon shanks [5,6] but never replicated for flexible arrays. We believe that edge electrodes have enhanced tissue contact and decreased distance to nearby neurons, improving the likelihood of single-unit recordings. This does not necessarily mean improved SNR or spike amplitude (Fig. S5-6). This geometric constraint may be the reason that other polymer arrays with electrodes placed near the center of wide, flexible shanks

reported a low yield of single-unit activity during NHP recordings [7–9]. These findings should strongly inform future design decisions for researchers developing neural interfaces.

Low drift Si recordings

Our flexible arrays in large animal models displayed dramatic reductions in relative probe drift. It should be noted that low-drift recordings with rigid arrays are possible. A recent study using Neuropixels 1.0 NHP reported a low drift similar to those we show here with flexible electrodes [10]. However, this stability also requires long “settling” periods after insertion lasting 30-60 min prior to recording, which is not always suitable for maintaining animal motivation during behavioral tasks. In addition, this study also used a blunt guide tube pressed against the brain surface to minimize motion and swelling. In our recordings, low drift was observed without a 30–60-min settling period or a blunt guide tube pressed against the brain surface.

SUPPLEMENTAL TABLES

Metric	Comparison	n1	n2	Median1	Median2	p	Significance
Max displacement	Plexon vs DBC	18	17	219.43	175	0.33643	ns
Max displacement	Plexon vs Flex	18	21	219.43	39.45	1.59E-06	***
Max displacement	Plexon vs Neuropixels	18	10	219.43	216.67	1	ns
Max displacement	DBC vs Flex	17	21	175	39.45	1.57E-05	***
Max displacement	DBC vs Neuropixels	17	10	175	216.67	0.33643	ns
Max displacement	Flex vs Neuropixels	21	10	39.45	216.67	2.35E-05	***
5-min drift	Plexon vs DBC	17	16	63.732	38.902	0.040104	*
5-min drift	Plexon vs Flex	17	21	63.732	7.45	3.1E-06	***
5-min drift	Plexon vs Neuropixels	17	10	63.732	69.156	0.93996	ns
5-min drift	DBC vs Flex	16	21	38.902	7.45	0.000126	***
5-min drift	DBC vs Neuropixels	16	10	38.902	69.156	0.057728	ns
5-min drift	Flex vs Neuropixels	21	10	7.45	69.156	7.84E-05	***

Table S1. Probe drift statistics including Neuropixels recordings.

Statistical results for session-level probe drift metrics across all probe types (combined across n = 2 animals). Each observation represents one independent recording session. Overall differences were assessed using two-sided Kruskal–Wallis tests, followed by two-sided Wilcoxon rank-sum tests with Bonferroni correction for multiple comparisons. Exact Bonferroni-adjusted P values are reported for every pairwise comparison in the table. Flexible probes exhibited significantly lower maximum displacement and 5-minute drift magnitude than Plexon, DBC, and Neuropixels probes, whereas Neuropixels recordings did not differ significantly from Plexon recordings. Values are reported as median session-level drift metrics.

SUPPLEMENTAL FIGURES

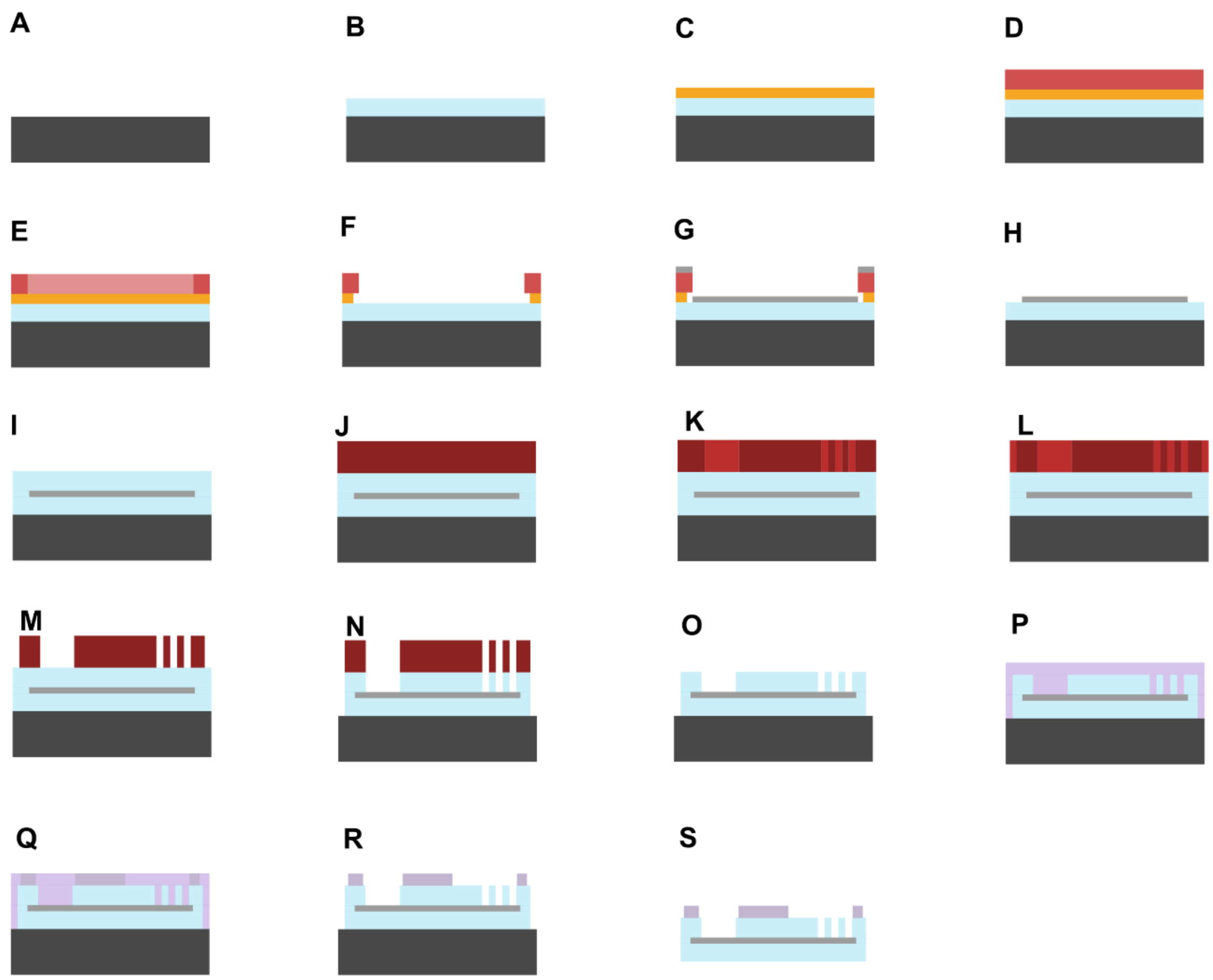

Figure S1: Full fabrication of flexible microelectrode arrays

(A) Si wafer substrate, (B) Parylene-C CVD, (C) LOR7A, (D) S1813, (E) Metal layer UV exposure, and (F) developing in MF-319, (G) Pt sputter deposition, (H) lift-off in remover 1165, (I) 2nd Parylene-C CVD, (J) SPR 7.0, (K) contact and connection pad UV exposure, (L) probe outline UV exposure, (M) probe electrode and outline development, (N) probe electrode and outline etching, (O) strip SPR 7.0, (P) coat in SU-8, (Q) UV expose probe tip and backend, (R) develop SU-8, (S) peel probe from Si substrate.

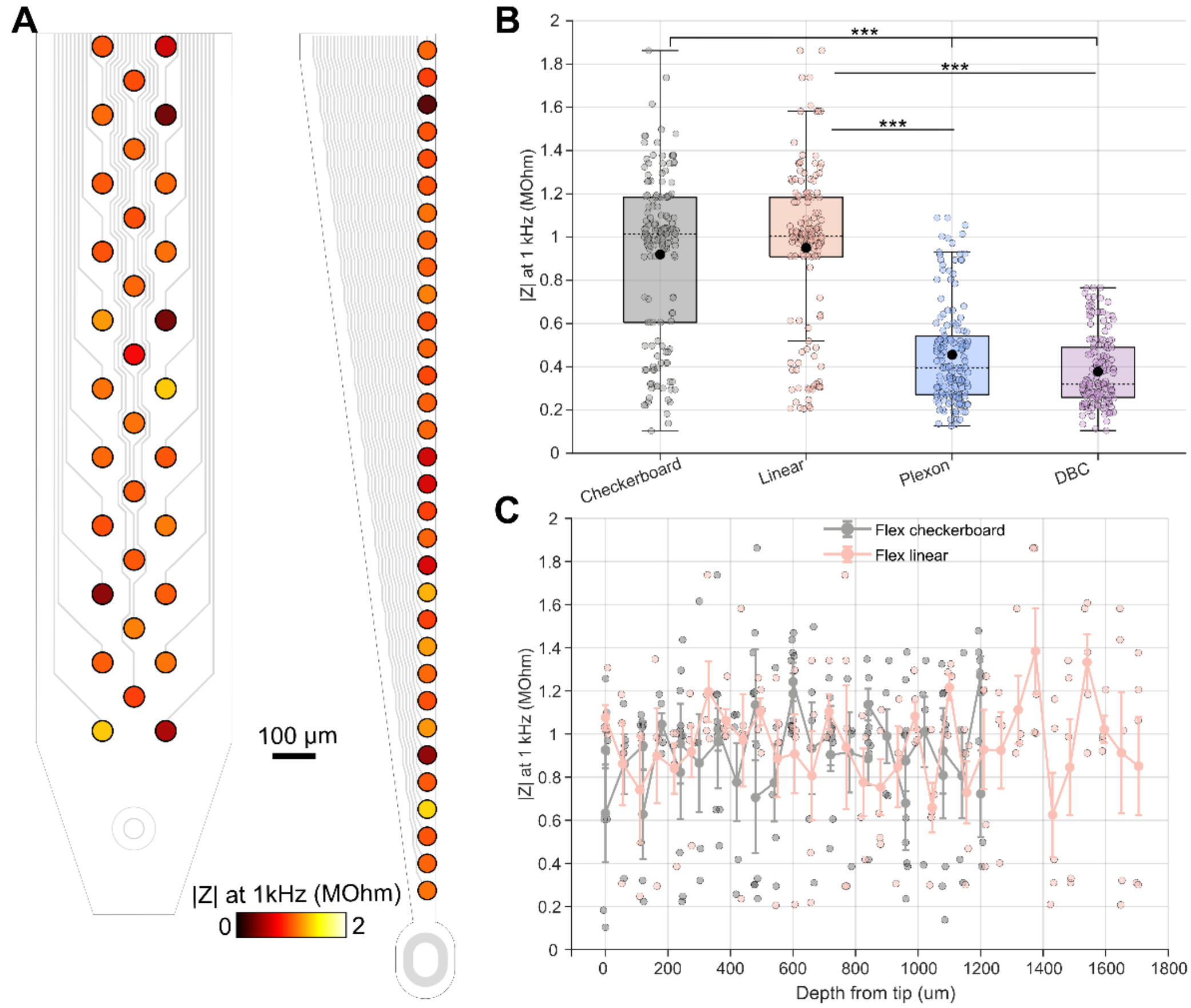

Figure S2: Impedance analysis of flexible and rigid arrays

(A) Heatmap of the 1 kHz electrochemical impedance for the two flexible array designs.

(B) Boxplots comparing the 1 kHz electrochemical impedance of recording sites from our two flexible array designs (checkerboard and linear edge), 32 Ch Plexon S/V probes, and 128 channel Diagnostic Biochips Deep Array Probes (n = 150, 157, 154, and 155 respectively). The center line indicates the median, the black circle indicates the mean, the box bounds indicate the 25th and 75th percentiles, whiskers extend to the most extreme data points within 1.5 × the interquartile range, and points beyond the whiskers are plotted individually as outliers.

(C) Scatter plot showing the 1 kHz electrochemical impedance of the flexible array designs as a function of distance from the probe tip. Flexible electrodes exhibited significantly higher impedance than both commercial rigid probe types (one-way ANOVA, $F(3,611) = 153.4$, $p < 0.0001$; Bonferroni-corrected post hoc comparisons, all $p < 0.0001$). Note that data shown here for flexible electrodes were recorded with a 3-point probe measurement, and all measurements from rigid electrodes were recorded using the impedance measurement function on the Intan RHD headstage (see Methods).

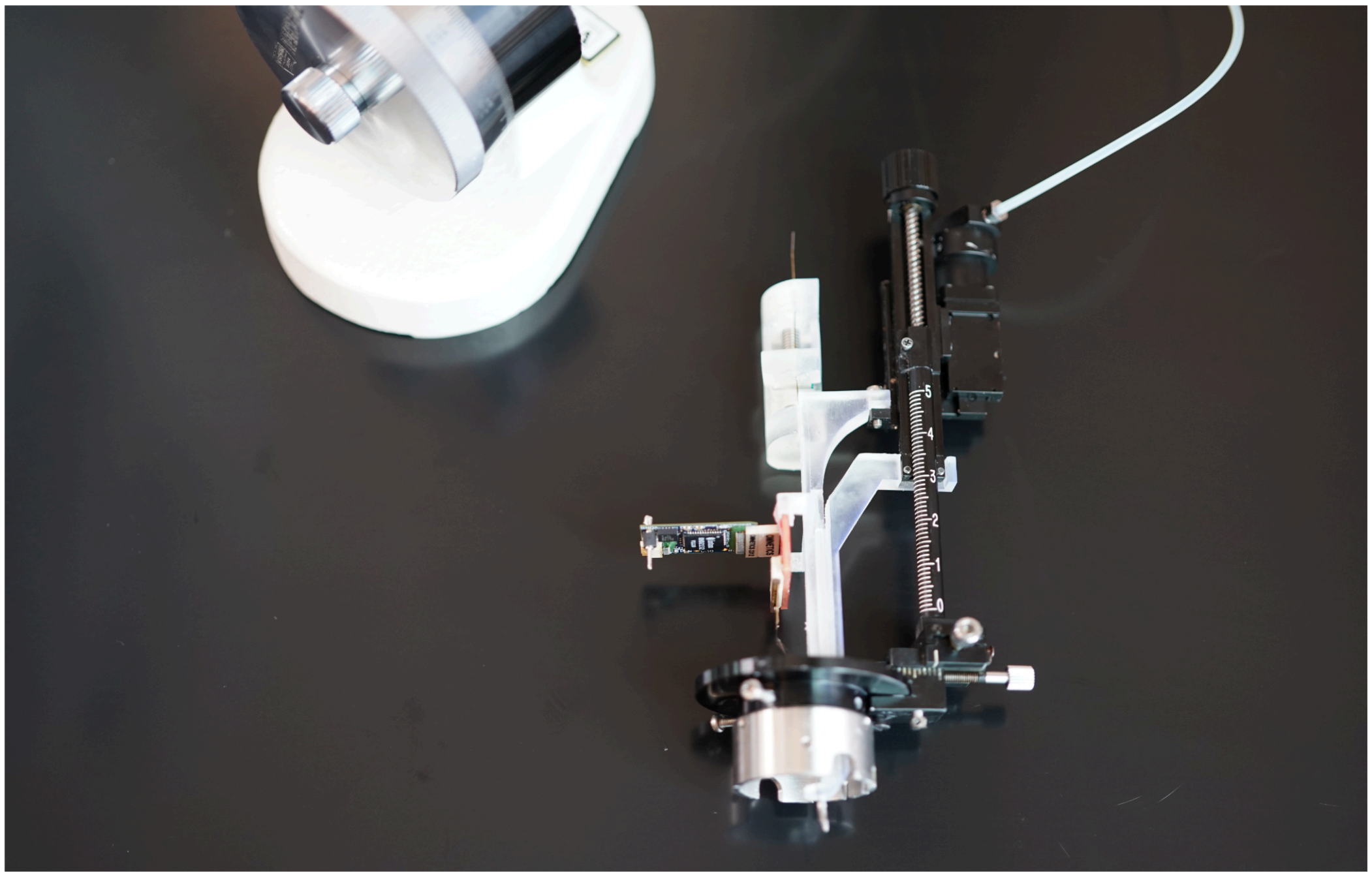

Figure S3: Microdrive adapter

Image shows Narishige MO-97A microdrive with our custom 3D printed drive adapter holding a loaded probe with the custom PCB and Intan headstage.

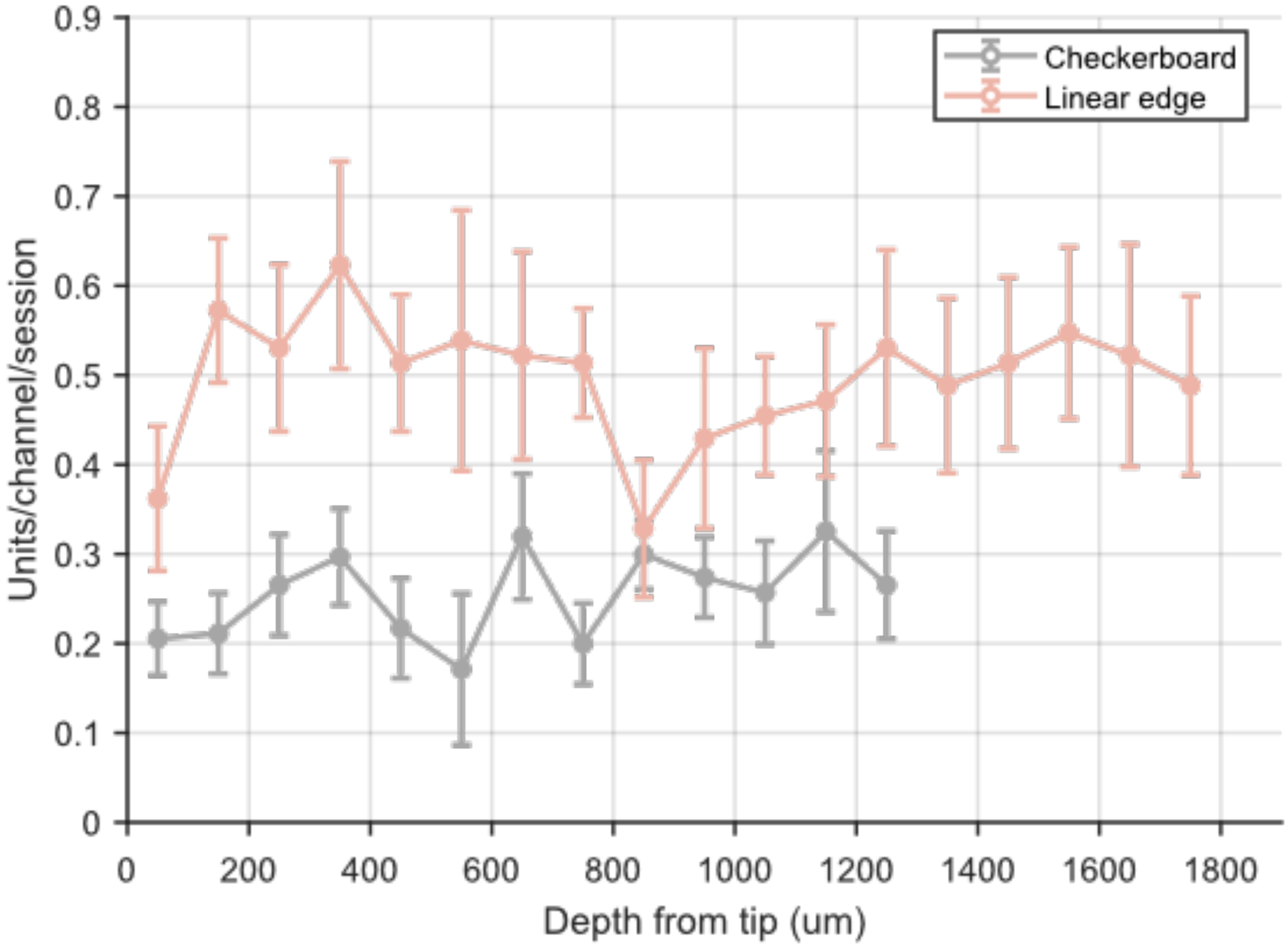

Figure S4. Flexible probe yield across depth.

Average recording-site yield (units/channel/session, mean ± SEM) as a function of distance from the probe tip for checkerboard and linear-edge flexible probes. Within-design comparisons revealed no significant depth dependence for either probe architecture: yield did not differ between the shallowest and deepest thirds of recording sites for the linear-edge design ($p = 0.886$) or the checkerboard design ($p = 0.195$). In contrast, the linear-edge design yielded significantly more units than the checkerboard design in both the shallowest third of recording sites ($p = 1.4 \times 10^{-8}$) and the deepest third of recording sites ($p = 6.5 \times 10^{-6}$).

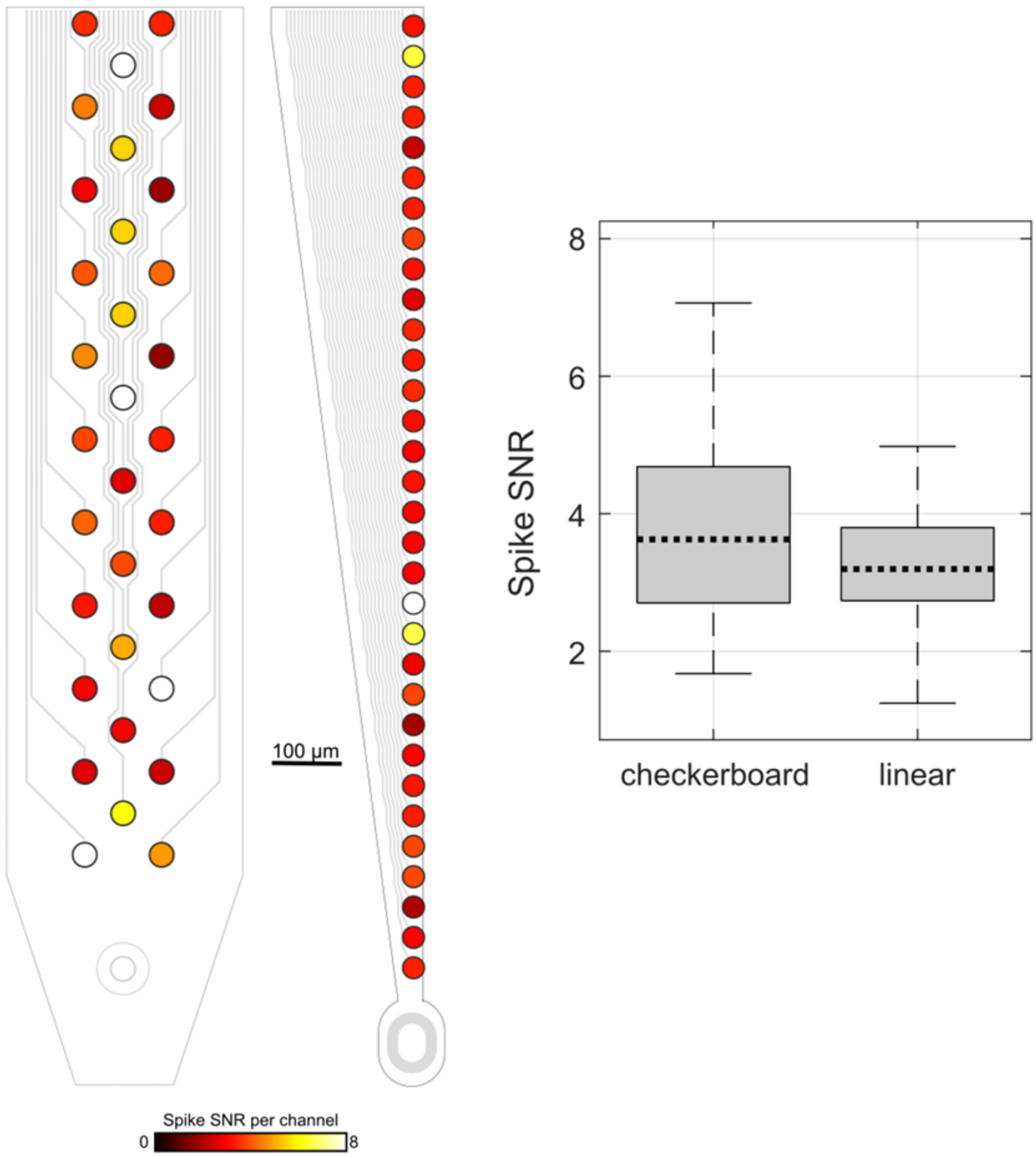

Figure S5: Signal-to-noise ratio between probe types

(Left and Center) Spike SNR per channel for both flexible array geometries, (Right) Median spike SNR across channel by probe type. The center line indicates the median, the box bounds indicate the 25th and 75th percentiles, and whiskers extend to the most extreme data points within 1.5 × the interquartile range.

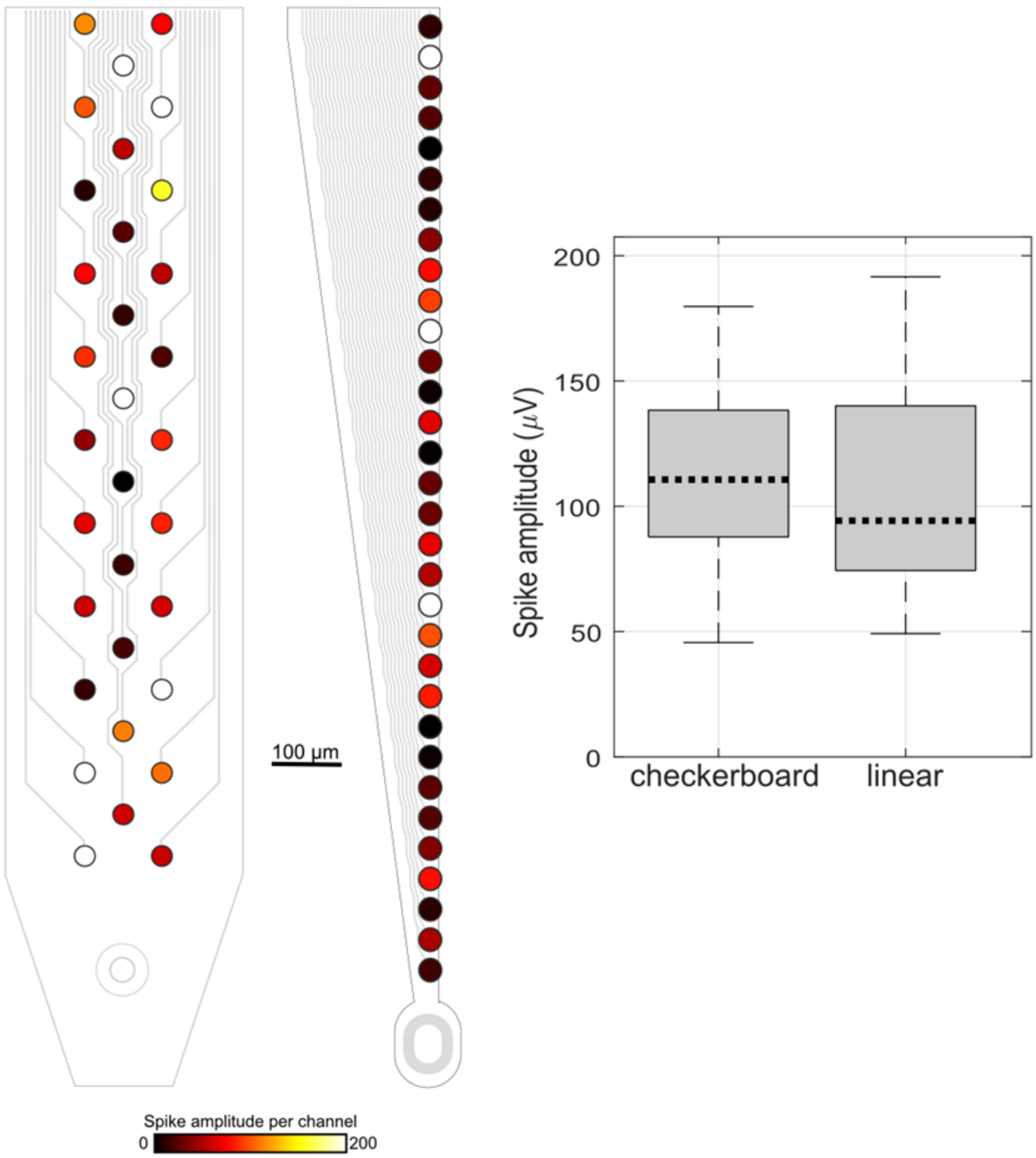

Figure S6: Unit amplitude between probe types

(Left and Center) Spike amplitude per channel for both flexible array geometries, (Right) Median spike amplitude across channels by probe type. The center line indicates the median, the box bounds indicate the 25th and 75th percentiles, and whiskers extend to the most extreme data points within 1.5 × the interquartile range.

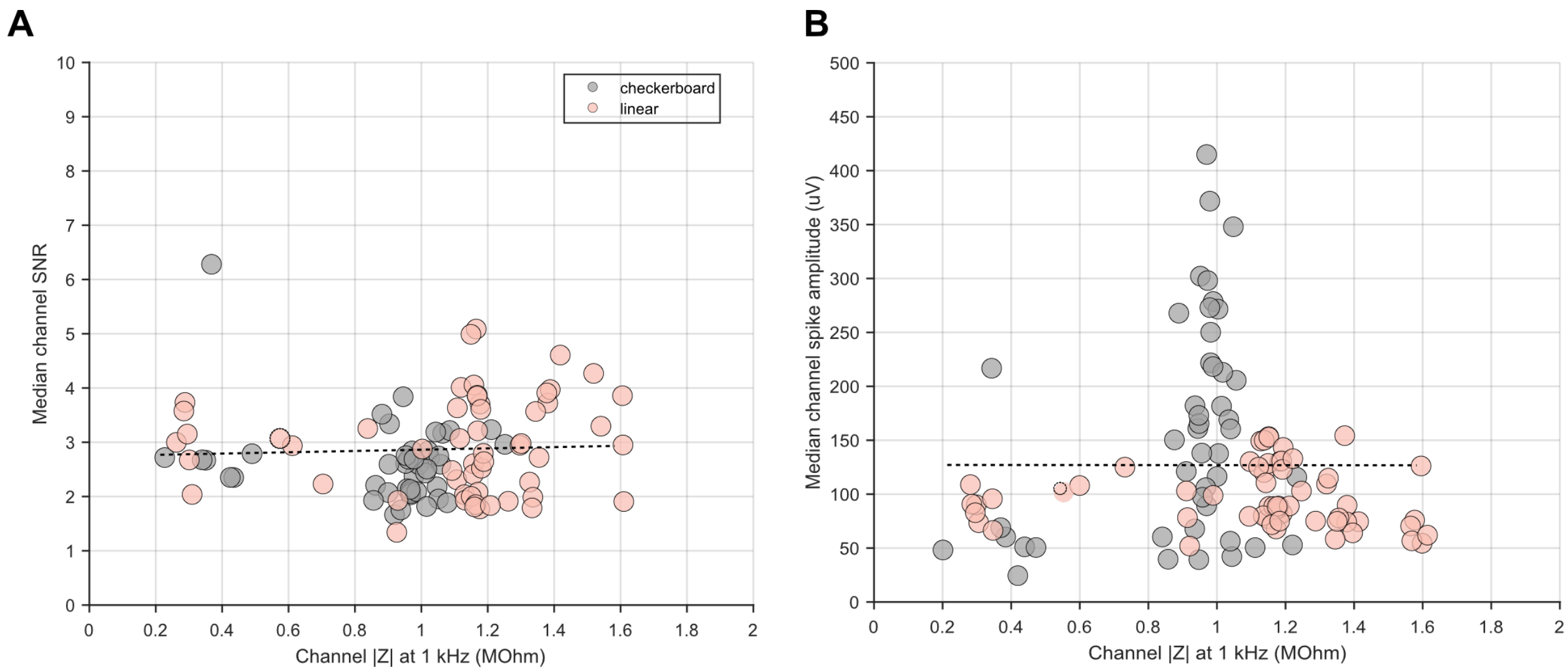

Figure S7: Signal-to-noise ratio versus contact impedance per site

(A) Median channel signal-to-noise ratio (SNR) plotted as a function of electrode impedance measured at 1 kHz for checkerboard and linear flexible array configurations. Each point represents a channel-session observation (n = 105 observations across 6 recording sessions and 46 recording sites). Electrode impedance was not significantly correlated with median channel SNR (Spearman $\rho = 0.144$, $p = 0.152$).

(B) Median channel spike amplitude plotted as a function of electrode impedance measured at 1 kHz for the same recording sites. Each point represents a channel-session observation. Electrode impedance was not significantly correlated with median channel spike amplitude (Spearman $\rho = -0.105$, $p = 0.289$).

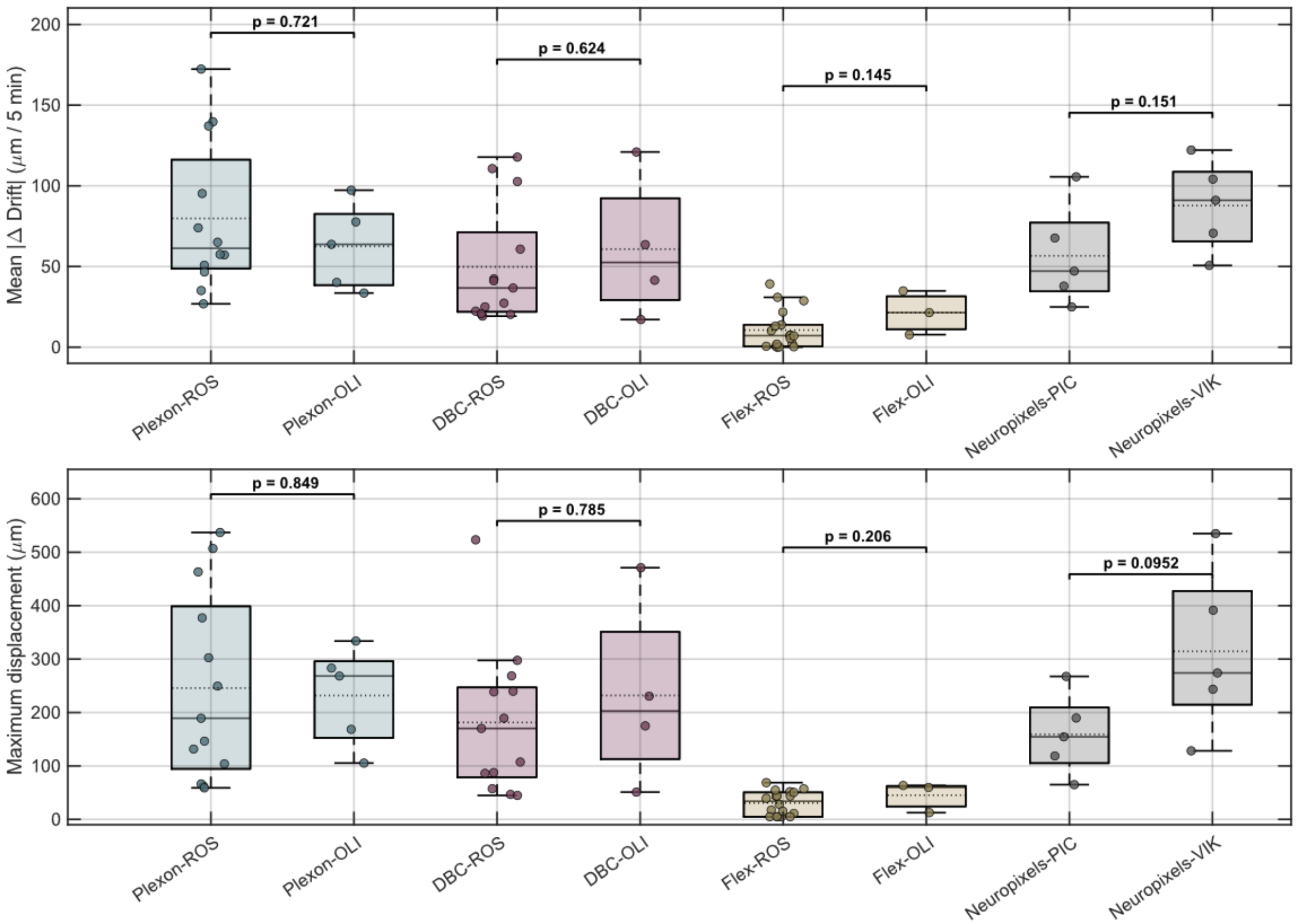

Figure S8. Flexible probes exhibit reduced drift across recording animals.
Animal separated distributions of probe drift metrics for Plexon, Diagnostic Biochips (DBC), flexible probes, and Neuropixels probes. Plexon, DBC, and flexible recordings were separated by animal identity (ROS and OLI), while Neuropixels recordings were separated by animal identity (PIC and VIK). (Top) Drift over 5-minute intervals. (Bottom) Maximum displacement. Individual points represent recording sessions, the center line indicates the median, the dotted line indicates the mean, the box bounds indicate the 25th and 75th percentiles, and whiskers extend to the most extreme data points within 1.5 × the interquartile range. No significant differences in 5-minute drift or maximum displacement were observed between animals within any probe type (all within-probe animal comparisons, $p > 0.05$, Wilcoxon rank-sum tests). In contrast, data compiled for both animals for each probe type showed that drift metrics for all rigid probes significantly differ from flexible probes (Table S1).

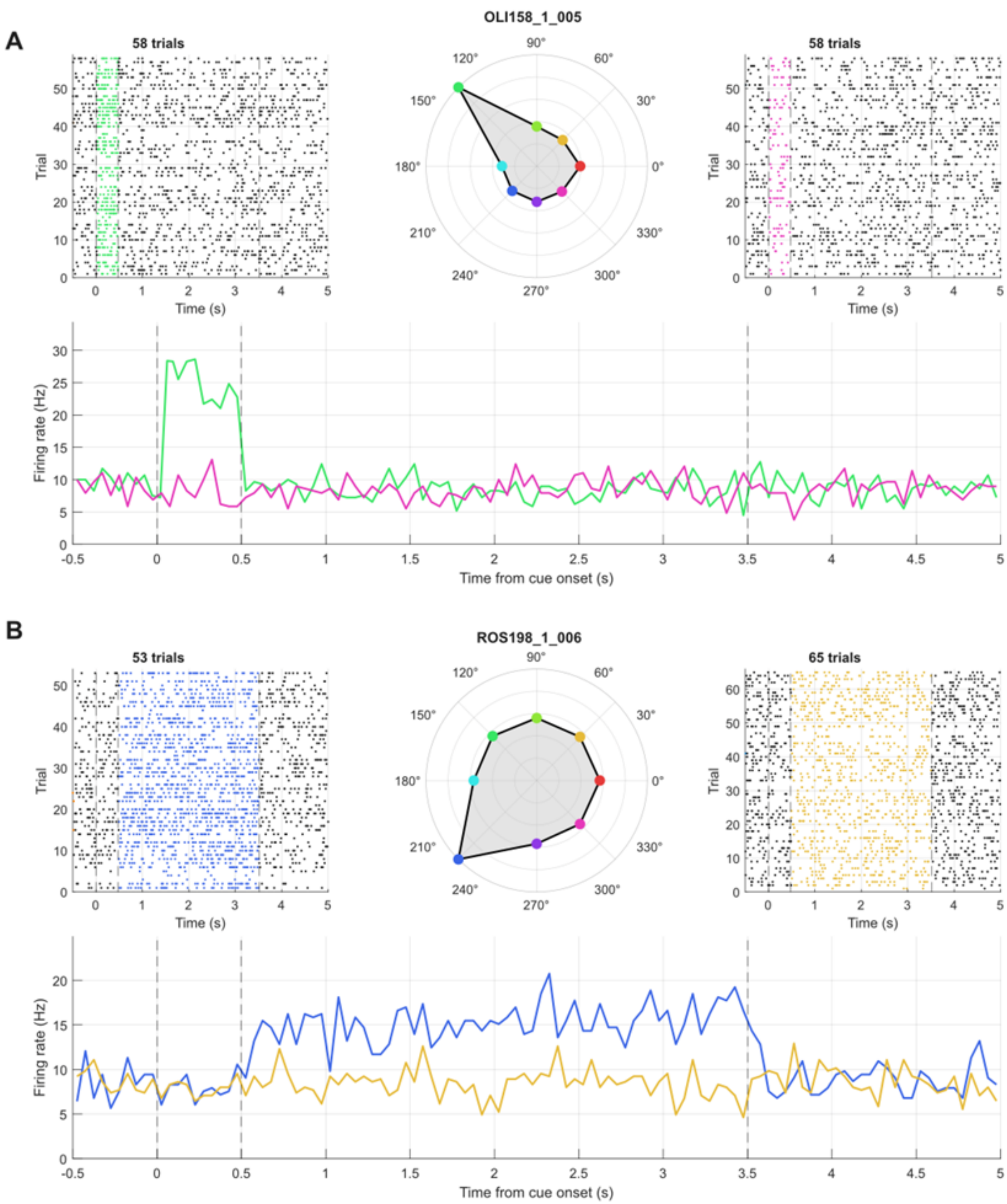

Figure S9: Representative cue and delay cells

(A) The same cells are shown in Fig. 5B. (Top left and right) Raster plots showing the preferred and diametric cue location for selected unit. (Top center) Polar plot showing preferred cue location for this cell. (Bottom) PSTH showing preferred (green) and opposite (cyan) traces for this cue responsive cell.

(B) (Top left and right) Raster plots showing the preferred and diametric cue location for selected unit. (Top center) Polar plot showing preferred cue location for this cell. (Bottom) PSTH showing preferred (blue) and diametric (yellow) traces for this delay responsive cell.

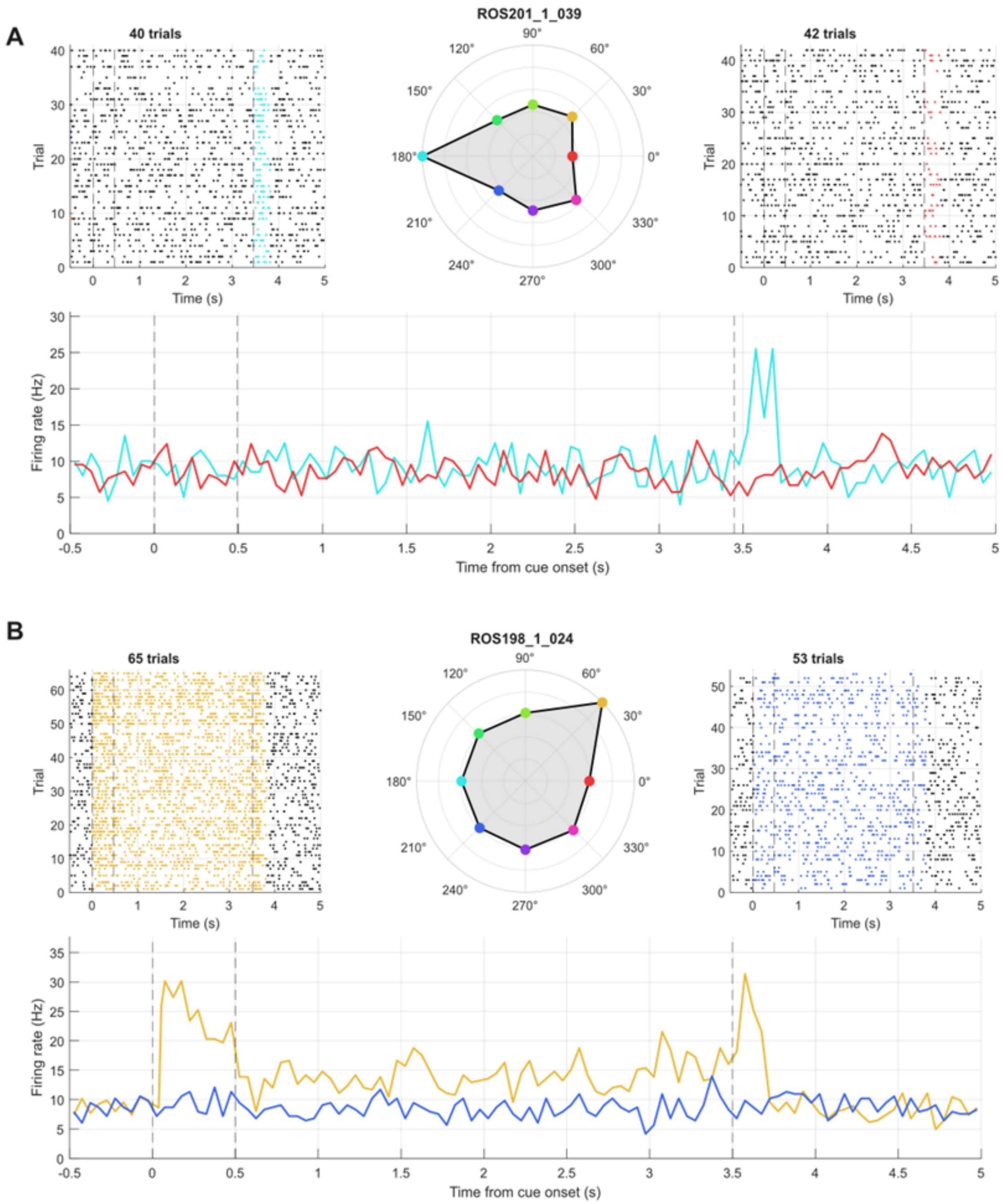

Figure S10: Representative saccade and multiple epoch cells

(A) (Top left and right) Raster plots showing the preferred and diametric cue location for selected unit. (Top center) Polar plot showing preferred cue location for this cell. (Bottom) PSTH showing preferred (blue) and diametric (red) traces for this saccade responsive cell.

(B) (Top left and right) Raster plots showing the preferred and diametric cue location for selected unit. (Top center) Polar plot showing preferred cue location for this cell. (Bottom) PSTH showing preferred (yellow) and diametric (blue) traces for this multi-epoch responsive cell.

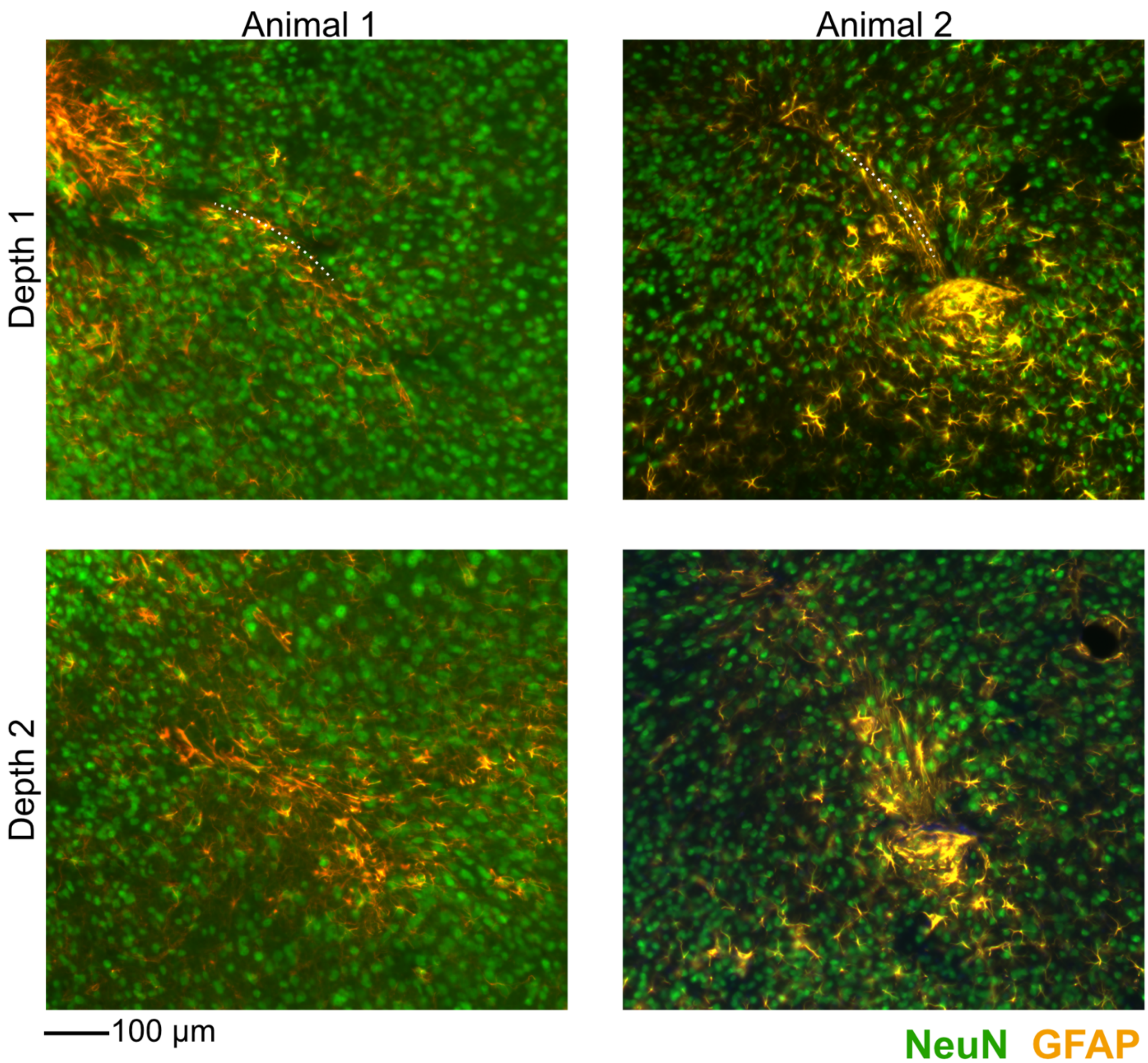

Figure S11: Immunohistochemistry of flexible probes implanted in mice.

Horizontal slices and staining from two animals with chronically implanted parylene and thin-film Pt microelectrodes. Depths are between 400 and 800 µm. The two depths shown are spaced by 240 µm. Animal 1 had an implant for 258 days and animal 2 for 178 days. Dotted line indicates the approximate location of the probe.

SUPPLEMENTARY REFERENCES

1. Luan, L. *et al.* Ultraflexible nanoelectronic probes form reliable, glial scar–free neural integration. *Sci. Adv.* **3**, e1601966 (2017).
2. Tian, Y. *et al.* An Ultraflexible Electrode Array for Large-Scale Chronic Recording in the Nonhuman Primate Brain. *Adv. Sci.* **10**, 2302333 (2023).
3. Wang, Y. *et al.* Flexible multichannel electrodes for acute recording in nonhuman primates. *Microsyst Nanoeng* **9**, 93 (2023).
4. Zhao, Z. *et al.* Parallel, minimally-invasive implantation of ultra-flexible neural electrode arrays. *J. Neural Eng.* **16**, 035001 (2019).
5. Fiáth, R. *et al.* Recording site placement on planar silicon-based probes affects signal quality in acute neuronal recordings. *Sci Rep* **11**, 2028 (2021).
6. Lee, H. C., Gaire, J., Roysam, B. & Otto, K. J. Placing Sites on the Edge of Planar Silicon Microelectrodes Enhances Chronic Recording Functionality. *IEEE Trans. Biomed. Eng.* **65**, 1245–1255 (2018).
7. Jeanpierre, G. M. *et al.* Flexible Microelectrode Arrays with Enhanced Electrochemical Properties Using Platinum Ditelluride. *ACS Appl. Electron. Mater.* **11, 7**(21) 9778–9791 (2025) doi:10.1021/acsaelm.5c01581.
8. Lee, K. *et al.* Flexible, scalable, high channel count stereo-electrode for recording in the human brain. *Nat Commun* **15**, 218 (2024).
9. Oh, S. *et al.* A stealthy neural recorder for the study of behaviour in primates. *Nat. Biomed. Eng* **9**, 882–895 (2025).
10. Trautmann, E. M. *et al.* Large-scale high-density brain-wide neural recording in nonhuman primates. *Nature Neuroscience* **28,** 1562–1575 (2025) doi:10.1038/s41593-025-01976-5.
