# Supplementary material for: Repeatable, low-drift recordings in behaving nonhuman primates using flexible microelectrodes
Source: Nat Commun. 2026 Sep 9;17:10638. doi: 10.1038/s41467-026-77421-3 (PMC13649665; doi:10.1038/s41467-026-77421-3)
Supplement: Supplementary file 2 [file 41467_2026_77421_MOESM2_ESM.pdf]

nature portfolio

Corresponding author(s): Daniel L., Gonzales

Last updated by author(s): Jul 30, 2026

Reporting Summary

Nature Portfolio wishes to improve the reproducibility of the work that we publish. This form provides structure for consistency and transparency in reporting. For further information on Nature Portfolio policies, see our Editorial Policies and the Editorial Policy Checklist.

Statistics

For all statistical analyses, confirm that the following items are present in the figure legend, table legend, main text, or Methods section.

n/a	Confirmed	
☐	☒	The exact sample size (n) for each experimental group/condition, given as a discrete number and unit of measurement
☐	☒	A statement on whether measurements were taken from distinct samples or whether the same sample was measured repeatedly
☐	☒	The statistical test(s) used AND whether they are one- or two-sided<br>*Only common tests should be described solely by name; describe more complex techniques in the Methods section.*
☐	☒	A description of all covariates tested
☐	☒	A description of any assumptions or corrections, such as tests of normality and adjustment for multiple comparisons
☐	☒	A full description of the statistical parameters including central tendency (e.g. means) or other basic estimates (e.g. regression coefficient) AND variation (e.g. standard deviation) or associated estimates of uncertainty (e.g. confidence intervals)
☐	☒	For null hypothesis testing, the test statistic (e.g. F, t, r) with confidence intervals, effect sizes, degrees of freedom and P value noted<br>*Give P values as exact values whenever suitable.*
☒	☐	For Bayesian analysis, information on the choice of priors and Markov chain Monte Carlo settings
☒	☐	For hierarchical and complex designs, identification of the appropriate level for tests and full reporting of outcomes
☐	☒	Estimates of effect sizes (e.g. Cohen's d, Pearson's r), indicating how they were calculated

Our web collection on statistics for biologists contains articles on many of the points above.

Software and code

Policy information about availability of computer code

Data collection	Recordings acquired with Open Ephys; Behavioral data acquired with Psychophysics Toolbox
Data analysis	Spike sorting performed with Kilosort 4; custom MATLAB scripts for analyses and recording drift are provided

For manuscripts utilizing custom algorithms or software that are central to the research but not yet described in published literature, software must be made available to editors and reviewers. We strongly encourage code deposition in a community repository (e.g. GitHub). See the Nature Portfolio guidelines for submitting code & software for further information.

Data

Policy information about availability of data

All manuscripts must include a data availability statement. This statement should provide the following information, where applicable:

- Accession codes, unique identifiers, or web links for publicly available datasets
- A description of any restrictions on data availability
- For clinical datasets or third party data, please ensure that the statement adheres to our policy

Data, code, probe layout files, and microdrive design files are publicly available at the repository: https://github.com/GonzalesLabVU/NHPflex2026

Research involving human participants, their data, or biological material

Policy information about studies with human participants or human data. See also policy information about sex, gender (identity/presentation), and sexual orientation and race, ethnicity and racism.

Reporting on sex and gender	n/a
Reporting on race, ethnicity, or other socially relevant groupings	n/a
Population characteristics	n/a
Recruitment	n/a
Ethics oversight	n/a

Note that full information on the approval of the study protocol must also be provided in the manuscript.

Field-specific reporting

Please select the one below that is the best fit for your research. If you are not sure, read the appropriate sections before making your selection.

☒ Life sciences ☐ Behavioural & social sciences ☐ Ecological, evolutionary & environmental sciences

For a reference copy of the document with all sections, see nature.com/documents/nr-reporting-summary-flat.pdf

Life sciences study design

All studies must disclose on these points even when the disclosure is negative.

Sample size	Samples sizes were not calculated prior to data collection
Data exclusions	Data from probes that broke upon implantation were not included
Replication	Replication was performed across n >= 10 neural data recording sessions
Randomization	The same animals were used for the primary comparisons in this manuscript. In this case, this is an attribute as metrics such as single-unit drift can vary across animals.
Blinding	No blinding was performed

Reporting for specific materials, systems and methods

We require information from authors about some types of materials, experimental systems and methods used in many studies. Here, indicate whether each material, system or method listed is relevant to your study. If you are not sure if a list item applies to your research, read the appropriate section before selecting a response.

Materials & experimental systems

n/a	Involved in the study	
☐	☒	Antibodies
☒	☐	Eukaryotic cell lines
☒	☐	Palaeontology and archaeology
☐	☒	Animals and other organisms
☒	☐	Clinical data
☒	☐	Dual use research of concern
☒	☐	Plants

Methods

n/a	Involved in the study	
☒	☐	ChIP-seq
☒	☐	Flow cytometry
☐	☒	MRI-based neuroimaging

Antibodies

Antibodies used	Rat anti-GFAP (Invitrogen 13-0300, RRID:AB_2532994, 1:1000). Alexa Fluor 647-conjugated rabbit anti-NeuN (Abcam ab190565, RRID:AB_2860568, 1:1000). Alexa Fluor 594 AffiniPure Goat Anti-Rat IgG (H+L) (Jackson ImmunoResearch 112-585-003, RRID:AB_2338372, 1:500).

Validation	Antibodies were selected based on prior validation by the manufacturers and established use in the neuroscience literature. The rat anti-GFAP antibody (Invitrogen 13-0300, clone 2.2B10, RRID:AB_2532994) recognizes glial fibrillary acidic protein and is validated by the manufacturer for immunohistochemistry in mouse tissue. The Alexa Fluor 647-conjugated rabbit anti-NeuN antibody (Abcam ab190565, clone EPR12763, RRID:AB_2732785) recognizes the neuronal nuclear antigen NeuN (Rbfox3) and is validated by the manufacturer for immunohistochemistry. The Alexa Fluor 594 AffiniPure Goat Anti-Rat IgG (H+L) secondary antibody (Jackson ImmunoResearch 112-585-003, RRID:AB_2338372) is validated by the manufacturer for immunofluorescence detection of rat primary antibodies. Antibody identities are additionally supported by their RRIDs.

Animals and other research organisms

Policy information about studies involving animals; ARRIVE guidelines recommended for reporting animal research, and Sex and Gender in Research

Laboratory animals	Two male, ~9 years old rhesus monkeys (Macaca mulatta) weighing 9-14 kg were used in this study for flexible, Plexon, and DBC electrode recordings (Animals O and R). Two additional animals (P and V) of similar age and sex were used for Neuropixels recordings. Monkeys were single housed in communal rooms, and they had sensory interactions with other monkeys. Mouse experiments were in adult Thy1-EGFP and Thy1-GCaMP6s animals (The Jackson Laboratory, #007788 and #024275, respectively). Mice were kept on a 12-hour light/dark cycle in conventional housing with unrestricted access to food and water.
Wild animals	n/a
Reporting on sex	Sex was not considered in this study do to the limitation of available animals.
Field-collected samples	n/a
Ethics oversight	All surgical and animal use procedures were reviewed and approved by the Vanderbilt University Institutional Animal Care and Use of Laboratory Animals and the National Research Council's Guide for the Care and Use of Laboratory Animals.

Note that full information on the approval of the study protocol must also be provided in the manuscript.

Plants

Seed stocks	n/a
Novel plant genotypes	n/a
Authentication	n/a

Magnetic resonance imaging

Experimental design

Design type	Anatomical only
Design specifications	n/a
Behavioral performance measures	n/a

Acquisition

Imaging type(s)	Structural
Field strength	3
Sequence & imaging parameters	natomical images were acquired using a T1-weighted magnetization-prepared rapid gradient-echo sequence: repetition time (TR)=2,700ms, echo time (TE)=3.32ms, inversion time=880, field of view (FOV)=128×128mm, 192 slices of 0.5-mm thickness, resolution=0.5mm isotropic. Resting state time series data were also acquired using a multiband echo-planar imaging sequence: TR=700ms, TE=32.0ms, flip angle=52°, repetitions=700, FOV=128×128mm, 32 slices, resolution=2mm isotropic.
Area of acquisition	whole brain

Diffusion MRI ☐ Used ☒ Not used

Preprocessing

Preprocessing software	We aligned anatomical T1 images to the high-resolution NIH Macaque Template (NMT) using @animal_warper (Jung et al., 2021; Saad et al., 2009). We used the 3D slicer software to find the projection of the recording cylinder on the brain surface, then localized electrode penetrations using the grid locations relative to the recording cylinder.
Normalization	n/a
Normalization template	n/a
Noise and artifact removal	n/a
Volume censoring	n/a

Statistical modeling & inference

Model type and settings	n/a
Effect(s) tested	n/a

Specify type of analysis: ☐ Whole brain ☐ ROI-based ☐ Both

Statistic type for inference (See Eklund et al. 2016)	n/a
Correction	n/a

Models & analysis

n/a	Involved in the study
☒	☐ Functional and/or effective connectivity
☒	☐ Graph analysis
☒	☐ Multivariate modeling or predictive analysis
