# Supplementary material for: Repeatable, low-drift recordings in behaving nonhuman primates using flexible microelectrodes
Source: Nat Commun. 2026 Sep 9;17:10638. doi: 10.1038/s41467-026-77421-3 (PMC13649665; doi:10.1038/s41467-026-77421-3)
Supplement: Supplementary file 3 [file 41467_2026_77421_MOESM3_ESM.pdf]

nature portfolio

Peer Review File

Repeatable, low-drift recordings in behaving non-human primates using flexible microelectrodes

Corresponding Author: Dr Daniel Gonzales

This file contains all reviewer reports in order by version, followed by all author rebuttals in order by version.

Version 0:

Reviewer comments:

Reviewer #1

(Remarks to the Author)
Summary
This manuscript presents a straightforward and well-executed study that bridges a significant gap between flexible microelectronics and large-animal neurophysiology. The authors demonstrate a comprehensive pipeline encompassing the design, microfabrication, and in vivo validation of a novel flexible electrode system specifically engineered for the macaque brain. Similar probes have been developed and used successfully in rodents and other small animals, but few other groups have been able to use “soft electrodes” to obtain high quality single unit recordings in monkeys. Indeed, the gold standard for multichannel neural recording in monkeys is to use intracranial chambers and rigid microelectrode arrays, which are vulnerable to signal drift due to the rigidity of the array relative to the brain.
The methodology is sound and meets the high standards of the field. The use of MRI/CT registration for cylinder placement and Kilosort for spike sorting follows best practices in NHP neurophysiology. The authors report some clever innovations, including the telescopic insertion shuttle, which is shown to enable effective penetration of NHP dura. The authors provide extensive detail in the Methods section, including specific fabrication parameters (Parylene-C thickness, SU-8 bake times), component part numbers (Hirose connectors, Intan headstages), and even 3D-printing plans for the custom microdrive. The commitment to making data, code, and probe layout files publicly available upon acceptance further ensures the work can be reproduced and adopted by the community.
This is a high-quality manuscript that provides a practical solution to a long-standing technical bottleneck in NHP neuroscience. I do have a few questions and suggestions that are intended as constructive feedback for the authors.
Comments and questions:
1. Scope and Terminology: Chronic vs. Acute Recordings
A critical distinction must be made regarding the study's scope versus its framing. The Abstract and Introduction frequently invoke the benefits of "chronic recordings" and "long-term stability." However, the experimental data presented focuses on acute, session-based recordings (1–2 hours) that are repeated over several weeks. While the system allows for repeatable access, it does not demonstrate the long-term, free-floating stability typically associated with the term "chronic" in the context of flexible electronics (where probes remain in the brain for months). The authors should either temper their claims to reflect that this is an optimized acute-delivery system that enables repeatability, or provide evidence of longitudinal single-unit tracking across multiple days without probe retraction.

2. Yield as a Function of Shank Geometry
The "Linear Edge" design is a notable improvement over the checkerboard layout, but its tapered geometry raises questions regarding differences in yield as a function of shank surface area. For example, the deepest electrodes occupy the narrowest portion of the shank, and it would be interesting to know if the yield is any different near the tip vs the base of the probe. The authors are encouraged to perform a depth-dependent yield analysis. This would clarify if the narrower tip (which causes less tissue displacement) provides a higher unit-per-channel ratio than the wider, more proximal portions of the shank. Performing the same analysis on the commercial steel electrodes—which have a uniform thickness—would serve as a necessary control to determine if yield differences are due to probe geometry or simply the laminar organization of the cortex.

3. Electrode impedances and SNR
Figure 1B shows single channel impedances range from 200 - 2000 kOhms; why is the variance so high? Does the

impedance vary with position along the shank? Did the authors measure changes in impedance before and after implant? The authors report a mean electrode impedance of 940 kΩ, which is fairly typical for microelectrodes of this type. Since the authors compare their probes to the COTS gold standards, it would be helpful to know the impedance properties of the COTS electrodes, in comparison to the flexible arrays. Are the impedances higher or lower? Is there any relationship between SNR and impedance?

4. Failure Analysis in the Experimental Pipeline
The flowchart provided in Figure 2 is an excellent guide for reproducibility; however, it lacks a few important details describing the nature of the failures. What was the location and cause of the 3 "breakages" that occurred during retraction? What was the nature of the 2 "hardware malfunctions"?

5. Is it possible to record from the electrodes during insertion or does the tungsten shuttle cause noise?
In many situations, it is helpful to record neural activity whilst actively navigating to a target. I'm curious if this system allows for this possibility.

(Remarks on code availability)

Reviewer #2

(Remarks to the Author)
This study addresses the signal drift caused by mechanical mismatch between rigid electrodes and soft brain tissue in non-human primate (NHP) neurophysiology by developing a 7 μm Parylene-C flexible microelectrode array and a "telescopic insertion" implantation system. It achieves repeated acute recordings in awake, behaving NHPs without the need for new craniotomies. It maybe the first quantitative, side-by-side comparison of single-unit drift between flexible and rigid electrodes in the same animals and chambers, showing that flexible electrodes reduce maximum drift from ~200 μm to ~40 μm. However, from a brain-computer interface (BCI) systems-integration perspective, the paper has notable gaps in chronic implant validation, histological assessment, control for systematic bias from different spike-sorting algorithms, and mechano-electrical modeling I recommend major revision prior to acceptance.

The abstract and title use phrases such as "repeatable, low-drift recordings" and "paves the way toward long-term, ultra-stable neurophysiology," yet all experiments are acute recordings. From a BCI and clinical translation perspective, acute and chronic recordings face fundamentally different biological challenges.

For a neural interface study, the complete absence of histological data (H&E, GFAP, Iba1, NeuN, etc.) is a major and unacceptable weakness. Given that a central claim of this work is that flexible electrodes reduce inflammation and glial scarring, the current argument relies entirely on electrophysiological inference without direct tissue-level evidence.

The Methods section states that flexible electrodes were sorted with Kilosort 4, whereas rigid electrodes were sorted with Kilosort 2.5. Could the"reduction in drift" be partially attributable to algorithmic improvements rather than purely hardware advantages?

The Discussion claims that the microfabrication process can be scaled to "hundreds of channels with no additional cost or time," and that a double-sided 128-channel shank is "feasible." However, the current system already uses a 32-channel Intan headstage and a ZIF connector of non-negligible size. As channel count increases, cable bundle thickness, connector size, mechanical load on the microwire shuttle, and the complexity of synchronizing multi-shank telescopic systems all scale non-linearly.

The rigid probes compared against the flexible arrays were Plexon S/V-Probes and DBC Deep Array Probes. These differ drastically in mechanical stiffness and cross-sectional dimensions. the current state-of-the-art benchmark in NHP neurophysiology is Neuropixels. If possible, the drift comparison with data from Neuropixels should be added.

(Remarks on code availability)

Version 1:

Reviewer comments:

Reviewer #1

(Remarks to the Author)
Thank you for the very thorough response to my comments, and congratulations on completing this exciting body of work.

(Remarks on code availability)

Reviewer #2

(Remarks to the Author)
The authors present a comprehensive methodology for acute, repeatable single-unit recordings in awake, behaving macaques.The core technical contribution is a "telescopic" insertion system that deploys free-floating flexible arrays through intact dura via standard chronic recording chambers, without new craniotomy or dura resection.Across 29 implantation attempts in two animals, the authors report an 82.8% penetration success rate and 87.5% shuttle-retraction success rate, with an 80% recording success rate after geometry optimization.However, generalizability is limited by 2 animals,

(Remarks on code availability)

Open Access This Peer Review File is licensed under a Creative Commons Attribution 4.0 International License, which permits use, sharing, adaptation, distribution and reproduction in any medium or format, as long as you give appropriate credit to the original author(s) and the source, provide a link to the Creative Commons license, and indicate if changes were made.
In cases where reviewers are anonymous, credit should be given to 'Anonymous Referee' and the source.
The images or other third party material in this Peer Review File are included in the article's Creative Commons license, unless indicated otherwise in a credit line to the material. If material is not included in the article's Creative Commons license and your intended use is not permitted by statutory regulation or exceeds the permitted use, you will need to obtain permission directly from the copyright holder.
To view a copy of this license, visit https://creativecommons.org/licenses/by/4.0/

Revision Summary

We would like to thank the reviewers for their rigorous and detailed reviews of our initial submission. In this Response, we include new recording data sets from NHPs, new histological data from months-long implants in mice, and extensive new analyses to address the key points raised. We believe these additions have strengthened the primary claims of the manuscript. Overall, the revised manuscript is significantly stronger, more rigorous, and better positioned to serve as a practical resource for investigators seeking to implement flexible neural interfaces in non-human primates. Please see our details point-by-point response in the following pages.

Reviewer #1

Summary

This manuscript presents a straightforward and well-executed study that bridges a significant gap between flexible microelectronics and large-animal neurophysiology. The authors demonstrate a comprehensive pipeline encompassing the design, microfabrication, and in vivo validation of a novel flexible electrode system specifically engineered for the macaque brain. Similar probes have been developed and used successfully in rodents and other small animals, but few other groups have been able to use "soft electrodes" to obtain high quality single unit recordings in monkeys. Indeed, the gold standard for multichannel neural recording in monkeys is to use intracranial chambers and rigid microelectrode arrays, which are vulnerable to signal drift due to the rigidity of the array relative to the brain.

The methodology is sound and meets the high standards of the field. The use of MRI/CT registration for cylinder placement and Kilosort for spike sorting follows best practices in NHP neurophysiology. The authors report some clever innovations, including the telescopic insertion shuttle, which is shown to enable effective penetration of NHP dura. The authors provide extensive detail in the Methods section, including specific fabrication parameters (Parylene-C thickness, SU-8 bake times), component part numbers (Hirose connectors, Intan headstages), and even 3D-printing plans for the custom microdrive. The commitment to making data, code, and probe layout files publicly available upon acceptance further ensures the work can be reproduced and adopted by the community.

This is a high-quality manuscript that provides a practical solution to a long-standing technical bottleneck in NHP neuroscience. I do have a few questions and suggestions that are intended as constructive feedback for the authors.

Comments and questions:

1. **Scope and Terminology: Chronic vs. Acute Recordings**

A critical distinction must be made regarding the study's scope versus its framing. The Abstract and Introduction frequently invoke the benefits of "chronic recordings" and "long-term stability." However, the experimental data presented focuses on acute, session-based recordings (1–2 hours) that are repeated over several weeks. While the system allows for repeatable access, it does not demonstrate the long-term, free-floating stability typically associated with the term "chronic" in the context of flexible electronics (where probes remain in the brain for months). The authors should either temper their claims to reflect that this is an optimized acute-delivery system that enables repeatability, or provide evidence of longitudinal single-unit tracking across multiple days without probe retraction.

As the reviewer suggested, we revised the main text in several locations (abstract, introduction, and discussion) to emphasize that our low-drift recordings were achieved in an acute context.

For example, in the abstract of the original submission: *"Our work establishes flexible microelectrode arrays as a practical, dependable technology for NHP neuroscience and paves the way toward long-term, ultra-stable neurophysiology in large animal models."*

Has been revised to: *"Our work establishes flexible microelectrode arrays as a practical, dependable technology for NHP neuroscience with clear recording stability advantages when compared to conventional electrodes."* (pg. 2)

Please see similar revisions in the abstract, introduction, and discussion as well, where we emphasize that these studies were conducted in acute settings. In the Discussion, we leave chronic implantations as a key area of future development.

2. Yield as a Function of Shank Geometry

The "Linear Edge" design is a notable improvement over the checkerboard layout, but its tapered geometry raises questions regarding differences in yield as a function of shank surface area. For example, the deepest electrodes occupy the narrowest portion of the shank, and it would be interesting to know if the yield is any different near the tip vs the base of the probe. The authors are encouraged to perform a depth-dependent yield analysis. This would clarify if the narrower tip (which causes less tissue displacement) provides a higher unit-per-channel ratio than the wider, more proximal portions of the shank. Performing the same analysis on the commercial steel electrodes—which have a uniform thickness—would serve as a necessary control to determine if yield differences are due to probe geometry or simply the laminar organization of the cortex.

As the reviewer suggested, we performed an additional analysis to determine whether the increased yield from our "linear edge" design was due to the edge-arranged electrodes, or the reduced probe width (275 µm that tapered to 50 µm, compared to our "checkerboard" design with a constant width of 340 µm). For both our flexible array designs, we found no significant difference in single-unit yield as a function of cortical depth (Fig. S4, see below). Rather, the linear edge design shows a steady ~2X increase in yield across the majority of the array compared to the checkerboard design. This strongly suggests that our higher measured yield was not due to the tapered width, but the arrangement of electrodes very near the probe edge. We have included these results in a new supplementary figure:

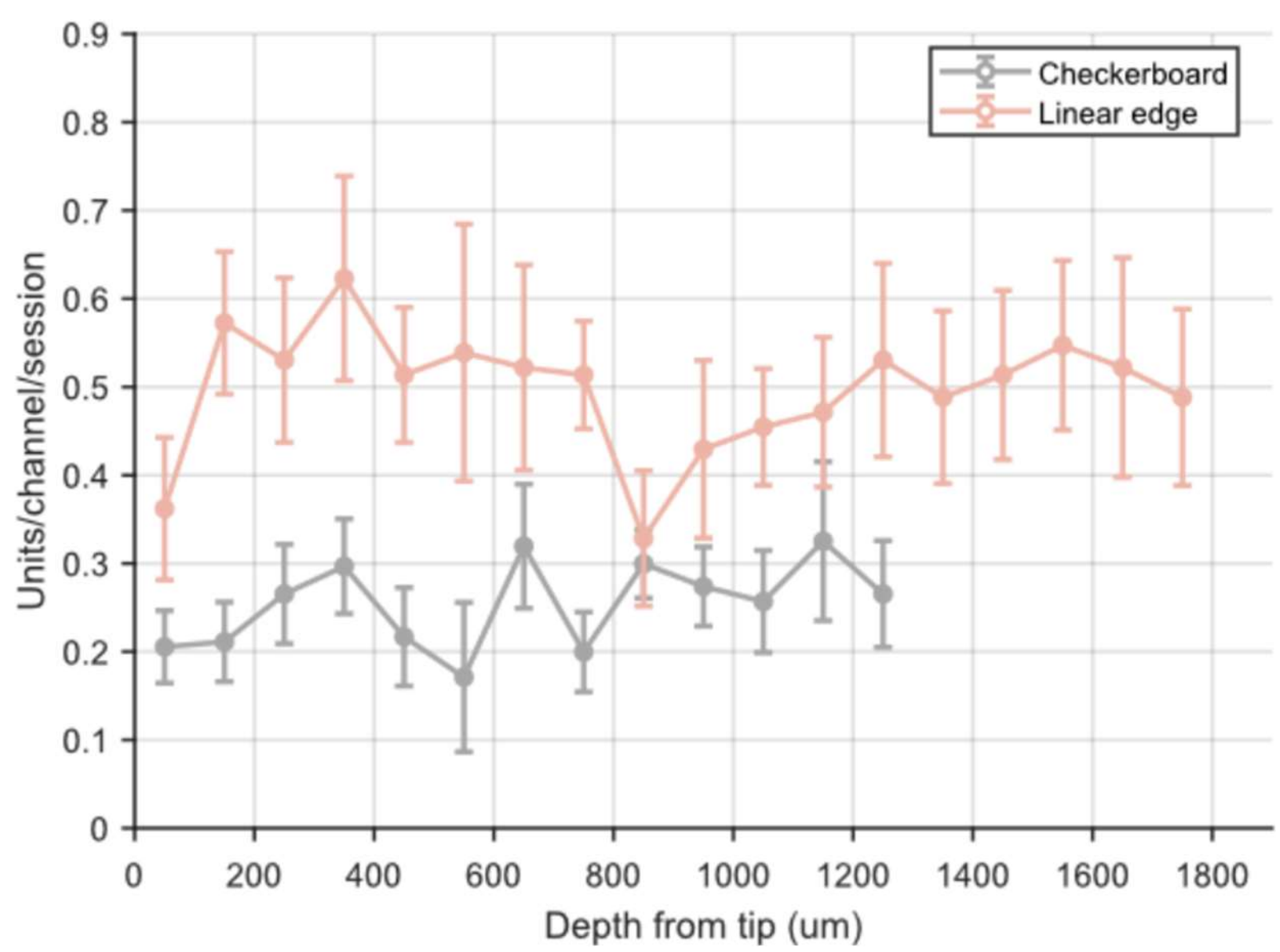

Figure S4. Flexible probe yield across depth.

Average recording-site yield (units/channel/session, mean ± SEM) as a function of distance from the probe tip for checkerboard and linear-edge flexible probes. Within-design comparisons revealed no significant depth dependence for either probe architecture: yield did not differ between the shallowest and deepest thirds of recording sites for the linear-edge design (p = 0.886) or the checkerboard design (p = 0.195). In contrast, the linear-edge design yielded

significantly more units than the checkerboard design in both the shallowest third of recording sites ($p = 1.4 \times 10^{-8}$) and the deepest third of recording sites ($p = 6.5 \times 10^{-6}$).

As requested, we also performed these depth analyses on commercial arrays, Plexon and DBC, which have a uniform probe length (200 μm diameter). Despite the geometric uniformity, the single-unit yield across the shanks does indeed vary (see below). However, comparison of single-unit yield as a function of depth in this manner becomes challenging. The rigid electrodes are longer, are inserted deeper, and span more cortical anatomy with likely differing neuronal densities. Therefore, it is unclear if the variable yield across depth for the rigid electrodes is due to probe performance or anatomical factors. Due to these confounding factors and challenging interpretation, we chose to not include this analysis in the new Figure S4 (see above) of the manuscript.

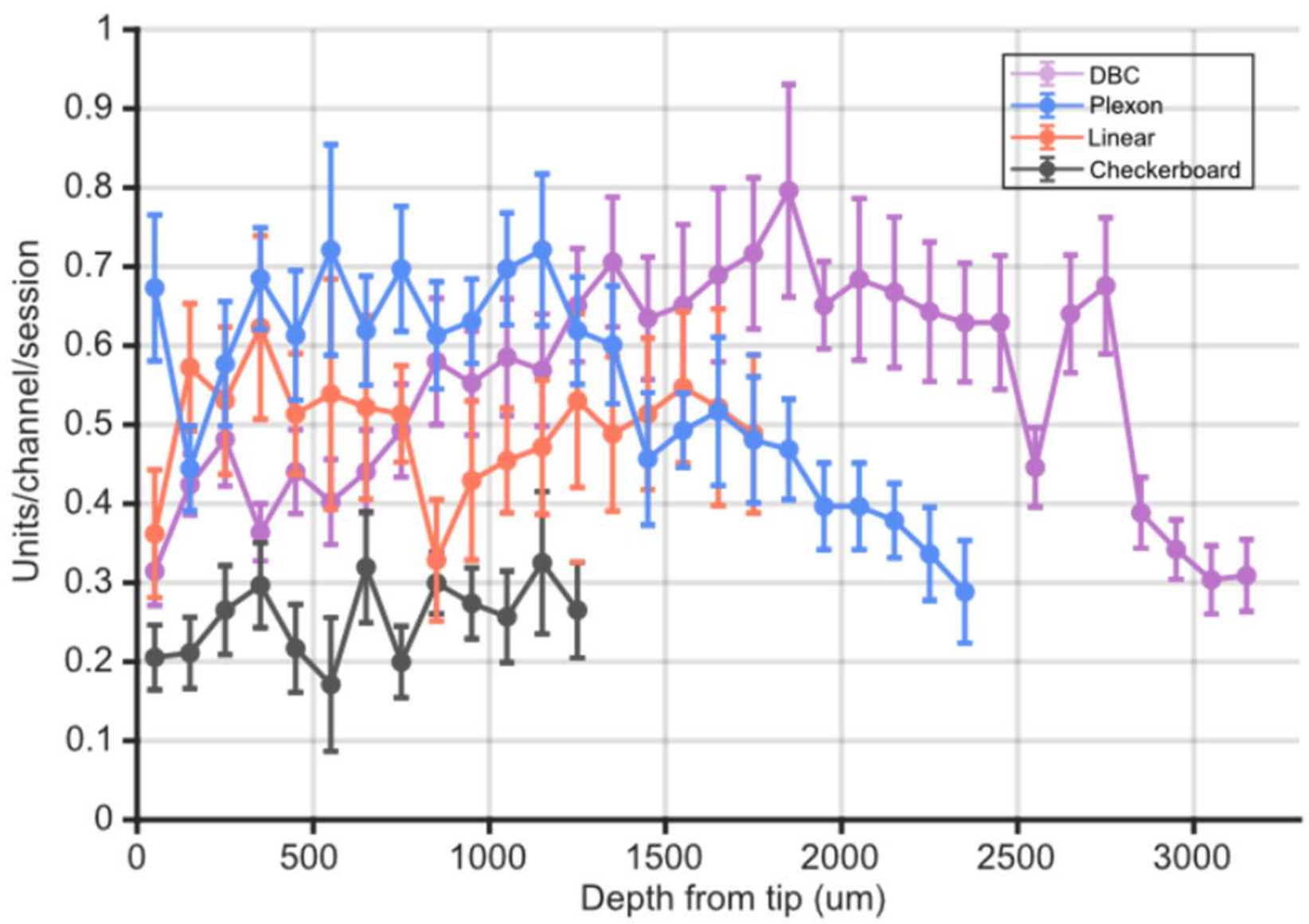

Figure: Unit yield across depth for all flexible and rigid probe types (not included in main manuscript)

Although we chose to not include the depth analysis for the rigid electrodes in the manuscript, we do believe an important takeaway for these new analyses is a comparison of the average single-unit yield for our flexible arrays and commercial electrodes. Across all recording sessions for flexible and rigid arrays, we computed the average single-unit yield per recording site. We found that our flexible linear edge design has a comparable single-unit yield to the rigid arrays and we have included these results in a main figure panel (see below, Fig. 3H).

Overall, these revisions strengthen some of our key claims: 1) our edge-arranged electrodes increased single-unit yield, 2) our linear edge arrays performed similarly to commercial electrodes, and 3) performance along the length of our flexible arrays is consistent.

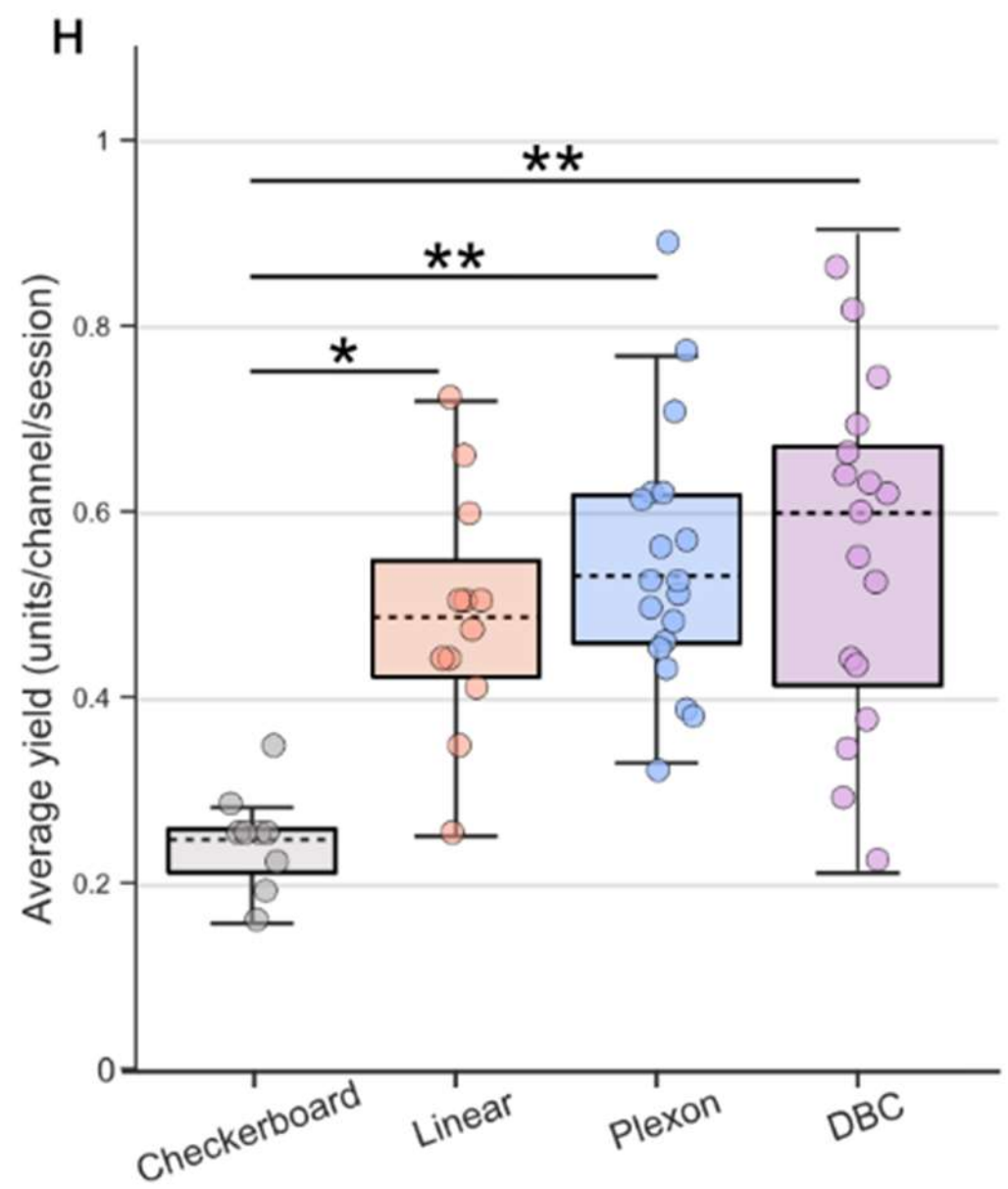

Figure 3. Flexible microelectrodes record single-unit activity across cortical regions.

(H) Boxplots show the distribution of units recorded per session for different probe types. Average recording-site yield differed significantly across probe types (one-way ANOVA, $F(3,57) = 6.14$, $p = 0.0011$). Linear flexible probes yielded 0.49 ± 0.04 units/channel/session, compared with 0.24 ± 0.02 units/channel/session for checkerboard probes, 0.55 ± 0.03 units/channel/session for Plexon probes, and 0.60 ± 0.06 units/channel/session for DBC probes (mean ± SEM). Post hoc Bonferroni-corrected comparisons showed that checkerboard probes yielded significantly fewer units/channel/session than linear flexible probes ($p = 0.017$), Plexon probes ($p = 0.0014$), and DBC probes ($p = 0.0018$), whereas linear flexible probes did not differ significantly from either Plexon or DBC probes (both $p = 1.0$).

In association with these figures, we have made the following changes to the main text:

“This increase was unlikely to result solely from the tapered geometry and reduced width of the linear-edge design near the probe tip. Although the linear edge design tapers from 275 µm to 50 µm, recording site yield did not vary significantly with depth (Fig. S4, $p = 0.195$). Instead, the linear design shows a steady yield across the length of the array when compared to the checkerboard design (Fig. S4, $p < 0.0001$)….. This provides substantial evidence that the design of flexible microelectrode arrays can influence recording quality.

Furthermore, we compared our single-unit yield results for flexible arrays to commercial rigid electrode arrays. In addition to the 21 recordings performed with flexible arrays in animals O and R, we performed 35 additional acute recordings in the same animals and chambers using a combination of 32 channel Plexon S/V-Probes and 128 channel Diagnostic BioChips (DBC) Deep Array Probes. Both rigid probes are fabricated from stainless steel with a 200-µm shank diameter and have an electrode spacing similar to our flexible devices. For all recordings, we sorted single units and found that the linear-edge design achieved single-unit yields comparable to both Plexon and Diagnostic Biochips probes units/channel/session (Welch's t-test, $p = 0.421$ and $p = 0.548$), while significantly outperforming the checkerboard geometry (0.25 ± 0.04 units/channel/session; $p = 0.0025$, Cohen's $d = 1.23$; Fig. 3H).” (pg. 10)

And, finally, changes to the abstract:

“Critically, by comparing our flexible arrays to rigid probes in the same animals and recording chambers, we provide quantitative evidence that flexible electrodes not only enable a similar single-unit yield but also reduce total single-unit drift from hundreds to tens of microns.” (pg. 2)

3. **Electrode impedances and SNR**

a. **Figure 1B shows single channel impedances range from 200 - 2000 kOhms; why is the variance so high?**

Commercial electrodes typically have an electrode coating (TiN, IrOx, PEDOT:PSS, etc) that minimizes the impedance variability from electrode to electrode. Our electrodes are bare platinum with no additional surface treatment. This likely leads to an overall higher variance in electrochemical performance compared to commercial electrodes. This unintentional variability provided the opportunity to evaluate whether a relationship exists between recording site electrochemical impedance and the single-unit yield or SNR. As we discuss in the next point, no such relationship existed. We added the following text to the Methods in the Flexible Microelectrode Fabrication section:

“We chose not to apply additional surface treatments to the Pt microelectrodes. Although this increased the variability of electrochemical impedance across recording sites (Fig. 1, Fig. S2), impedance was not significantly correlated with channel SNR or spike amplitude (Fig. S7).” (pg. 19)

b. **Does the impedance vary with position along the shank?**

We performed additional benchtop electrochemical impedance measurements for a total of n = 10 arrays and 307 electrodes (Fig. S2 A&C, see below). We quantified the relationship between impedance and electrode depth and found that impedance did not vary systematically along the length of either flexible probe shank. We added the following text to the main manuscript:

“Each recording site had a diameter of 30 µm, yielding a ~706 µm² contact area and mean electrochemical impedance at 1 kHz of approximately 935 +/- 21 kΩ (Fig 1B), with no significant association between the electrochemical impedance and recording site position along the arrays (Fig. S2).” (pg. 5)

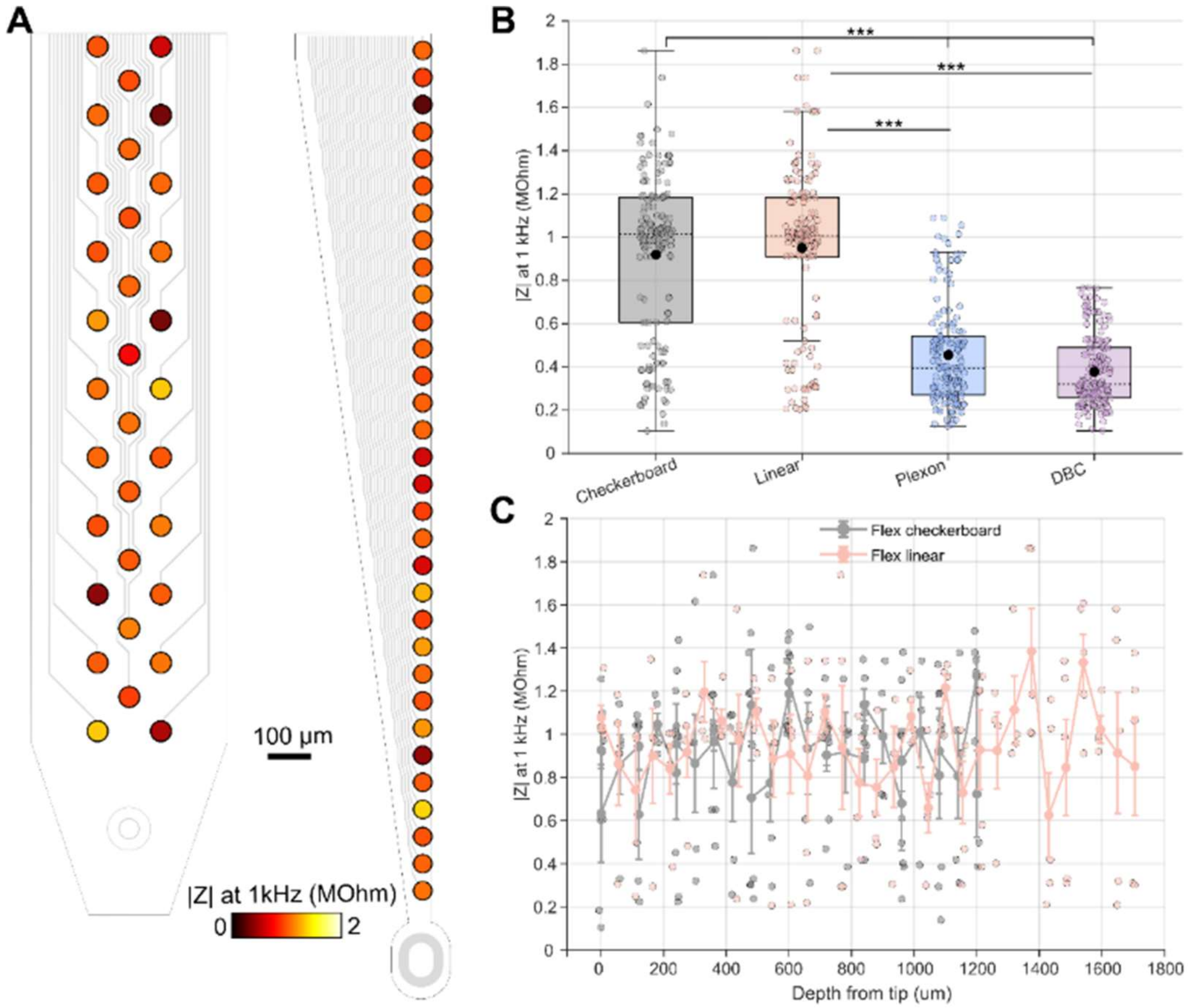

Figure S2: Impedance analysis of flexible and rigid arrays

(A) Heatmap of the 1 kHz electrochemical impedance for the two flexible array designs.

(B) Boxplots comparing the 1 kHz electrochemical impedance of recording sites from our two flexible array designs (checkerboard and linear edge), 32 Ch Plexon S/V probes, and 128 channel Diagnostic Biochips Deep Array Probes (n = 150, 157, 154, and 155 respectively).

(C) Scatter plot showing the 1 kHz electrochemical impedance of the flexible array designs as a function of distance from the probe tip. Flexible electrodes exhibited significantly higher impedance than both commercial rigid probe types (one-way ANOVA, $F(3,611) = 153.4$, $p < 0.0001$; Bonferroni-corrected post hoc comparisons, all $p < 0.0001$). Note that data shown here for flexible electrodes were recorded with a 3-point probe measurement, and all measurements from rigid electrodes were recorded using the impedance measurement function on the Intan RHD headstage (see Methods).

c. Did the authors measure changes in impedance before and after implant?

We did not perform these measurements.

d. The authors report a mean electrode impedance of 940 kΩ, which is fairly typical for microelectrodes of this type. Since the authors compare their probes to the COTS gold standards, it would be helpful to know the impedance properties of the COTS electrodes, in comparison to the flexible arrays. Are the impedances higher or lower?

We performed a 1 kHz electrochemical impedance measurement on COTS electrodes (DBC and Plexon) and compared the results to our flexible arrays. Our two flexible probe designs exhibited indistinguishable impedance at 1 kHz, whereas both commercial probes had significantly lower impedances (Fig. S2B, see above). We updated the main text and Electrochemical Impedance section of the methods accordingly:

Main text: *"Each recording site had a diameter of 30 µm, yielding a ~706 µm² contact area and mean electrochemical impedance at 1 kHz of approximately 935 +/- 21 kΩ (Fig 1B), with no significant association between the electrochemical impedance and recording site position along the arrays (Fig. S2). Comparatively, we measured the 1 kHz impedance for two commercial rigid arrays, 32 channel Plexon S/V probes and 128 channel Diagnostics BioChips Deep Array probes, which had average values of 455 +/- 19 kΩ and 378 +/- 13 kΩ, respectively (Fig. S2)." (pg. 5)*

Methods: *"For flexible microelectrodes, we tested benchtop impedance using three-electrode electrochemical impedance spectroscopy in PBS saline (Gamry Interface 1010E, 10 mV RMS amplitude, 1-10,000 Hz sweep). The recording site was used as the working electrode, an Ag/AgCl electrode as the reference electrode, and Pt wire as the counter electrode. For commercial rigid electrodes, the reported 1 kHz electrochemical impedances were recorded using on-chip measurements with the Intan RHD headstage and submerging the probe in saline with respect to an Ag/AgCl electrode."* (pg. 20)

e. Is there any relationship between SNR and impedance?

We performed additional analyses to determine if the measured benchtop 1 kHz electrochemical impedance was related to the flexible electrode single-unit recording. For single-unit yield and SNR, we did not see a clear relationship (Fig. S7, see below). We have added the following main text and supplementary figure:

"Both array designs exhibited a statistically identical 1 kHz electrochemical impedance (Fig. S2) and we observed no clear relationship between the recording site impedance and median channel SNR ($\rho = 0.144$, $p = 0.152$) or spike amplitude ($\rho = -0.105$, $p = 0.289$) (Fig. S7, n = 6 sessions, 105 recording sites)." (pg. 10)

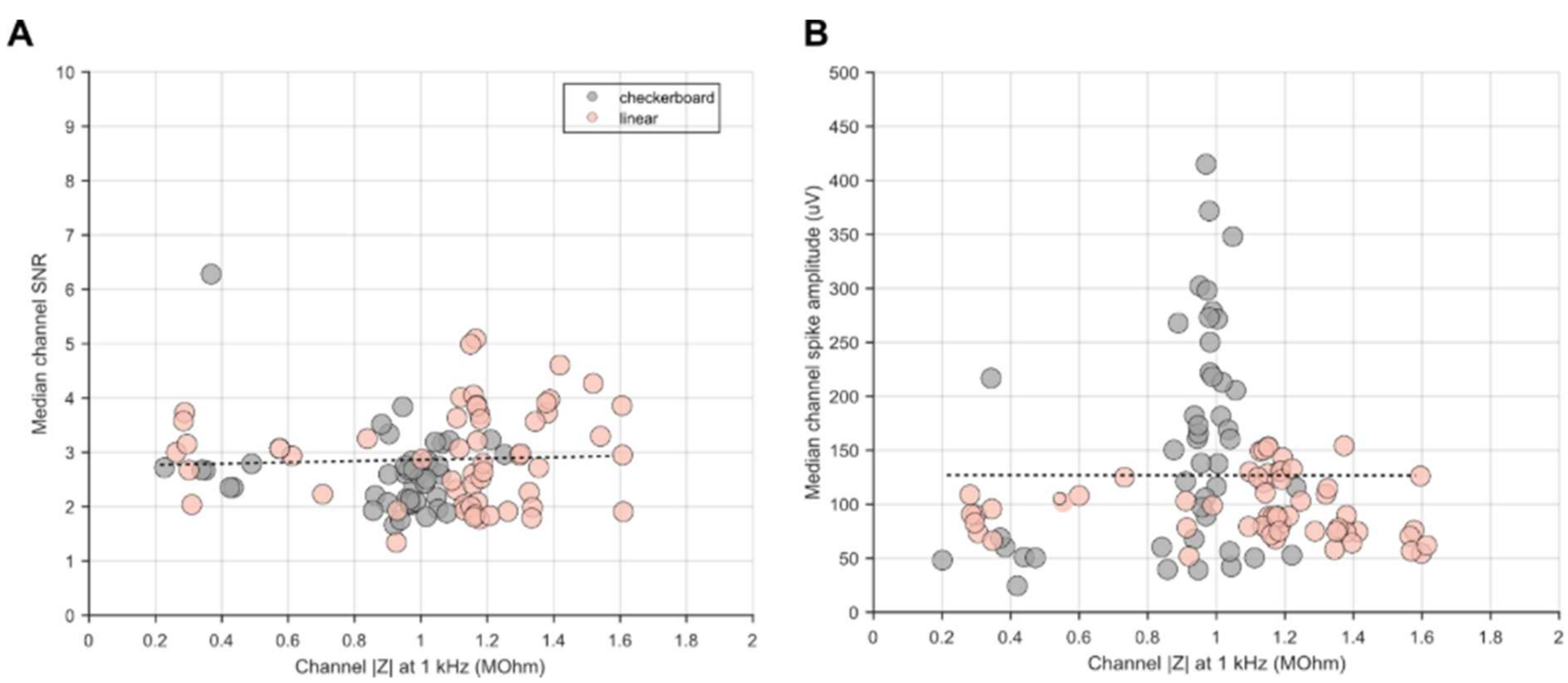

Figure S7: Signal-to-noise ratio versus contact impedance per site

(A) Median channel signal-to-noise ratio (SNR) plotted as a function of electrode impedance measured at 1 kHz for checkerboard and linear flexible array configurations. Each point represents a channel-session observation (n = 105 observations across 6 recording sessions and 46 recording sites). Electrode impedance was not significantly correlated with median channel SNR (Spearman ρ = 0.144, p = 0.152).

(B) Median channel spike amplitude plotted as a function of electrode impedance measured at 1 kHz for the same recording sites. Each point represents a channel-session observation. Electrode impedance was not significantly correlated with median channel spike amplitude (Spearman ρ = −0.105, p = 0.289).

4. **The flowchart provided in Figure 2 is an excellent guide for reproducibility; however, it lacks a few important details describing the nature of the failures. What was the location and cause of the 3 "breakages" that occurred during retraction? What was the nature of the 2 "hardware malfunctions"?**

We have added the following to the main text to better describe the "breakage" that occurred during implantation:

"In 29 in vivo attempts in two animals, we successfully penetrated to the recording depth in 24 sessions (82.8%, Fig. 2D, Table 1). Retraction was also highly reliable. Our flexible arrays remained in the brain in 87.5% of successful penetrations (21 sessions total, Fig. 2D, Table 1). In all successful retractions, we also recorded single-unit activity from the free-floating array (Fig. 2D). Retractions that failed (n = 3), were due to the tungsten wire tearing the thread-hole of the flexible array, most like during the advancement into tissue (Fig. 2)." (pg. 7)

We also added the following to describe the hardware malfunctions that affected behavioral monitoring during recordings:

"From 21 neural recording sessions, 18 also contained sufficient behavioral data during execution of this task (Fig. 2D). In the three failed 3 sessions, recordings were obtained, but due to hardware malfunctions we were unable to correlate electrophysiology with behavior (Fig. 2D, n = 1 eye tracking software failure during recording, n = 2 eye tracking hardware failure)." (pg. 14)

We made slight changes to Fig. 2D to better represent the "no behavior" recordings:

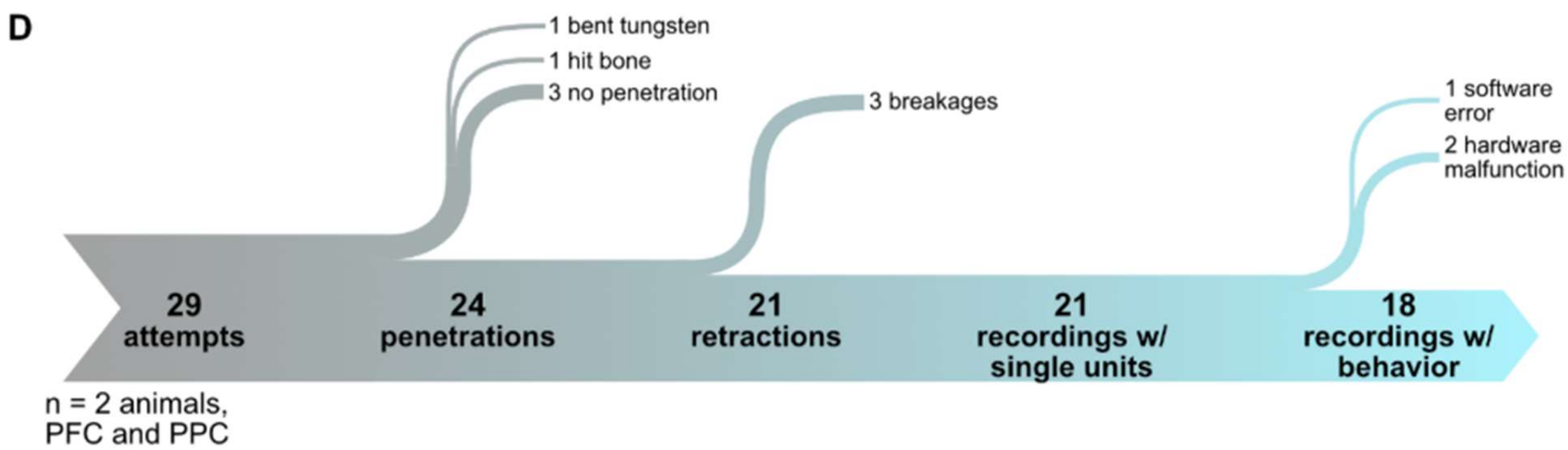

5. **Is it possible to record from the electrodes during insertion or does the tungsten shuttle cause noise? In many situations, it is helpful to record neural activity whilst actively navigating to a target. I'm curious if this system allows for this possibility.**

While it is possible to record during insertion, in most cases we do not. The tungsten wire is grounded to reduce any potential noise. The biggest factor is that we added a small amount of PEG (a dissolvable adhesive) to temporarily adhere the flexible array and tungsten wire. This facilitates insertion. Unfortunately, the PEG usually prevents quality recordings during the initial insertion period until it dissolves away (~5 min). Other groups have reported insertion with only threading, and not PEG. In the future, this would be ideal to target specific depths and brain regions by recording during insertion. We have added the following to the Methods to clarify this point:

"Threading of the flexible array occur prior to implantation. We first pre-routed the tungsten microwire through microdrive and 23 G support tube, then threaded the microwire tip to the flexible array and temporarily attached the two with the assistance of poly-ethylene glycol (PEG). We aligned the microwire such that it protruded from the support tube by approximately 5 mm. During insertion, we estimate it takes approximately 5 minutes for the PEG to fully dissolve. During this period, recordings are temporarily inhibited. This makes it challenging to record during the insertion process." (pg. 21)

Reviewer #2

Summary

This study addresses the signal drift caused by mechanical mismatch between rigid electrodes and soft brain tissue in non-human primate (NHP) neurophysiology by developing a 7 μm Parylene-C flexible microelectrode array and a "telescopic insertion" implantation system. It achieves repeated acute recordings in awake, behaving NHPs without the need for new craniotomies. It maybe the first quantitative, side-by-side comparison of single-unit drift between flexible and rigid electrodes in the same animals and chambers, showing that flexible electrodes reduce maximum drift from ~200 μm to ~40 μm. However, from a brain-computer interface (BCI) systems-integration perspective, the paper has notable gaps in chronic implant validation, histological assessment, control for systematic bias from different spike-sorting algorithms, and mechano-electrical modeling. I recommend major revision prior to acceptance.

1. The abstract and title use phrases such as "repeatable, low-drift recordings" and "paves the way toward long-term, ultra-stable neurophysiology," yet all experiments are acute recordings. From a BCI and clinical translation perspective, acute and chronic recordings face fundamentally different biological challenges.

Throughout the main text (abstract, introduction, and discussion) we made revisions that emphasize that our low-drift recordings were achieved in an acute context and that chronic implants are a key area of future work.

For example, in original abstract: *"Our work establishes flexible microelectrode arrays as a practical, dependable technology for NHP neuroscience and paves the way toward long-term, ultra-stable neurophysiology in large animal models."*

Has been revised to: *"Our work establishes flexible microelectrode arrays as a practical, dependable technology for NHP neuroscience with clear recording stability advantages when compared to conventional electrodes."* (pg. 2)

Please see similar revisions in the introduction and discussion.

In addition, we also made revisions that better represent the challenges ahead for chronic recordings. In the Discussion, we present chronic recordings as a clear next step of our work, but that these require come important considerations, particularly from a biological standpoint:

"However, the chronic integration of flexible microelectrodes in NHPs poses unique challenges that must be considered. For example, the long-term inflammatory response to implanted flexible arrays in large-brained animals remains poorly documented. In rodents, it is well established that thin, flexible electrodes implanted on the order of months integrate well with neural tissue, demonstrated by healthy neurons within microns of the array with minimal aggregation of microglia and reactive astrocytes (Chung, Joo, Fan, et al., 2019; Kim et al., 2013; Lu et al., 2016; Luan et al., 2017; Lycke et al., 2023; Musk & Neuralink, 2019; Yasar et al., 2024; S. Zhao et al., 2023; Z. Zhao et al., 2022; Zhou et al., 2017) Indeed, when implanted for more than 175 days in mice, our own flexible electrodes also show minimal neuronal displacement and comparable astrocyte aggregation near the implantation site (Fig. S11). Unfortunately, similar replications in NHPs with flexible electrodes, while promising, remain sparse and largely unquantified (Tian et al., 2023). Additional studies are required to assess whether flexible electrodes provide i) biological stability, ii) materials stability, and iii) recording stability in months- and years-long contexts in large-brained animals. Our work with acute recordings here suggests that the recording stability offered by flexible electrodes, paired with the possibility of minimal immune response when chronically implanted, may yield excellent long-term recording performance in NHPs." (pg. 17-18)

2. For a neural interface study, the complete absence of histological data (H&E, GFAP, Iba1, NeuN, etc.) is a major and unacceptable weakness. Given that a central claim of this work is that flexible electrodes reduce inflammation and glial scarring, the current argument relies entirely on electrophysiological inference without direct tissue-level evidence.

To address this, we have revised the abstract, the main text, and also added histological data. Our primary claim in this manuscript is that flexible microelectrodes increase recording stability in NHPs by reducing signal drift when compared to rigid electrodes. Therefore, we have revised the text to address inadvertent suggestions that our microelectrodes also reduce glial scarring in NHPs.

For example, in the original abstract: *"While these devices enable high-quality recordings, a fundamental mechanical mismatch between rigid materials and soft brain tissue leads to inflammation, gliosis, and signal instability."*

Revised submission: *"While these devices enable high-quality recordings, a fundamental mechanical mismatch between rigid materials and soft brain tissue leads to signal instability."* (pg. 2)

See similar changes to the introduction, where we focus the discussion on brain micromotion and signal instability, rather than gliosis and scarring from electrode implantation.

Additionally, we have added mouse histological data. Performing histological analyses on the macaques used in the study is not feasible. These animals are currently undergoing further study and duration of penetrations with our flexible arrays may not be sufficient to assess the tissue response. Therefore, we implanted our parylene microelectrodes in mice for extended time periods (>175 days) and performed histology. We stained for neuronal cell bodies (NeuN) and astrocytes (GFAP) and observed neuronal bodies near the implanted array and astrocyte aggregation that is comparable to other studies (Fig. S11, see below). We reference this data in the Discussion, where we present chronic implantation as an important next step in our technological development and where tissue integration becomes a critical factor in recording performance:

"In rodents, it is well established that thin, flexible electrodes implanted on the order of months integrate well with neural tissue, demonstrated by healthy neurons within microns of the array with minimal aggregation of microglia and reactive astrocytes (Chung, Joo, Fan, et al., 2019; Kim et al., 2013; Lu et al., 2016; Luan et al., 2017; Lycke et al., 2023; Musk & Neuralink, 2019; Yasar et al., 2024; S. Zhao et al., 2023; Z. Zhao et al., 2022; Zhou et al., 2017) Indeed, when implanted for more than 175 days in mice, our own flexible electrodes also show minimal neuronal displacement and comparable astrocyte aggregation near the implantation site (Fig. S11)." (pg. 17-18)

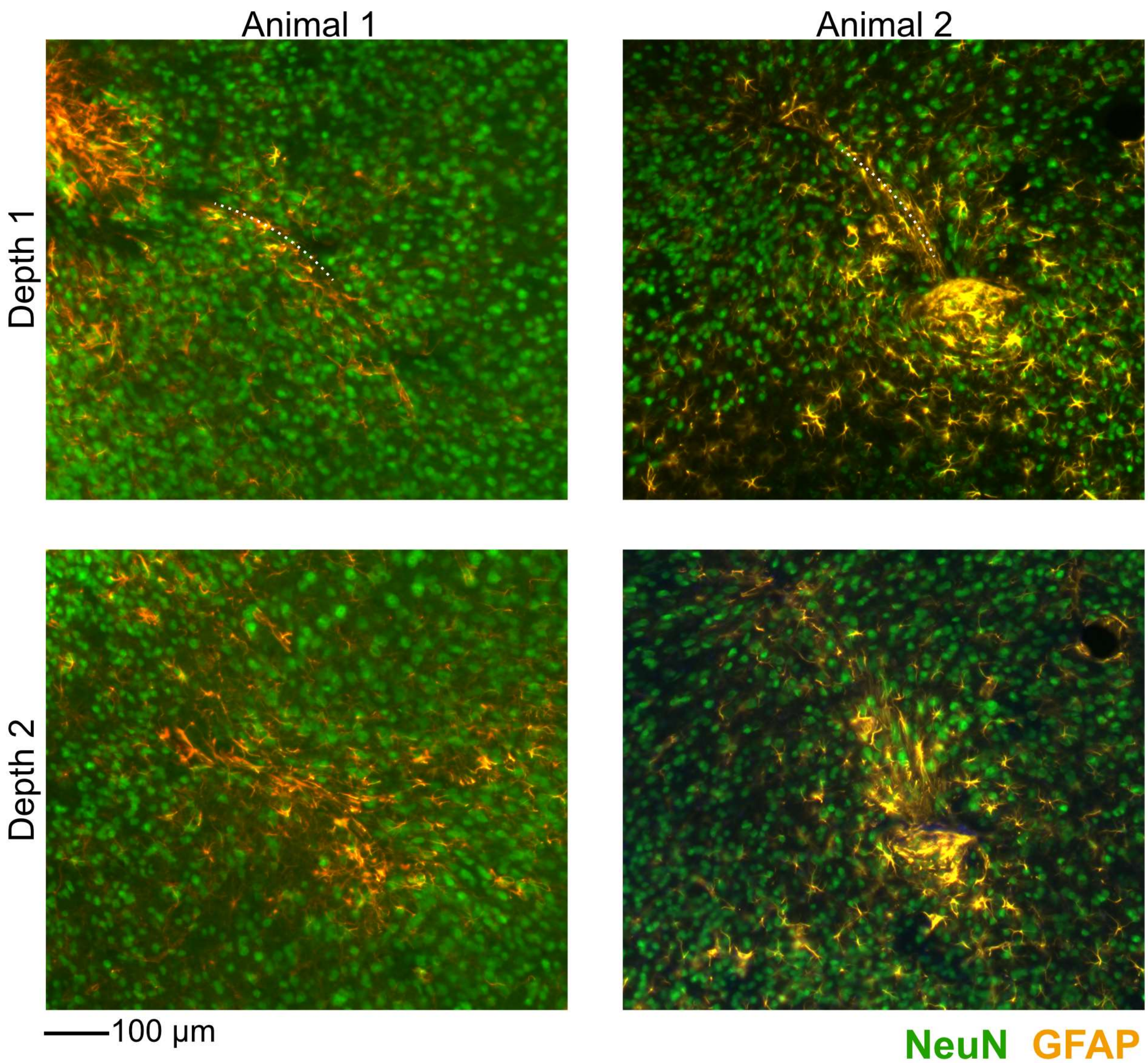

Figure S11: Immunohistochemistry of flexible probes implanted in mice.

Horizontal slices and staining from two animals with chronically implanted parylene and thin-film Pt microelectrodes. Depths are between 400 and 800 µm. The two depths shown are spaced by 240 µm. Animal 1 had an implant for 258 days and animal 2 for 178 days. Dotted line indicates the approximate location of the probe.

3. The Methods section states that flexible electrodes were sorted with Kilosort 4, whereas rigid electrodes were sorted with Kilosort 2.5. Could the "reduction in drift" be partially attributable to algorithmic improvements rather than purely hardware advantages?

We reprocessed all recordings using Kilosort 4 and repeated the drift analysis using the new outputs. Our key results and claims remain unchanged—our flexible electrodes substantially lowered the single-unit drift (Fig. 4, see below). All previous results from Kilosort 2.5 have been removed.

We updated the Methods section accordingly: *"We identified spike waveforms and sorted single units using the automated spike-sorting software Kilosort 4"* (pg. 23, Methods "Neural Data Processing" section)

We also have updated the main text and Fig. 4 appropriately to present only the analyses with Kilosort 4:

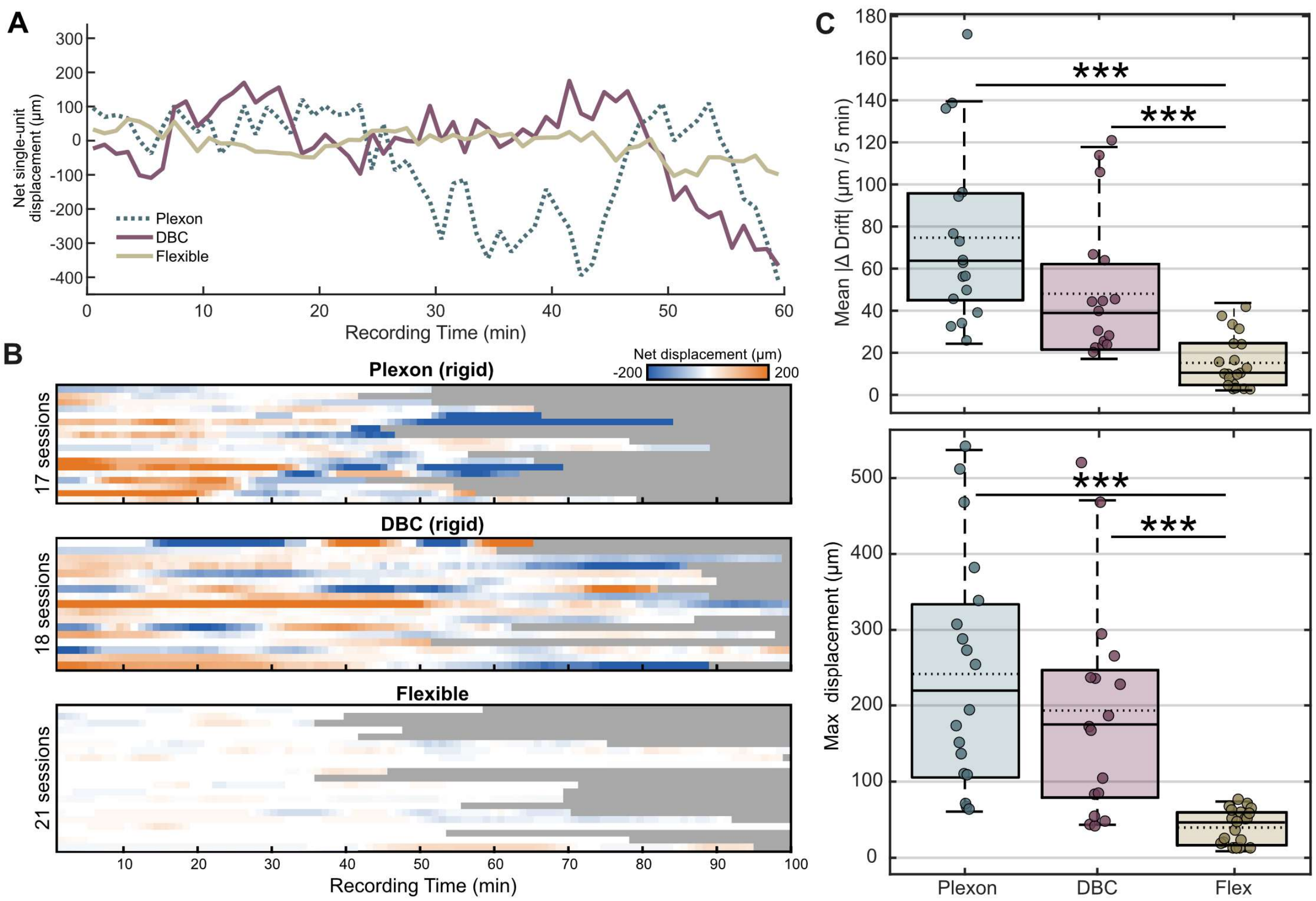

Figure 4. Flexible arrays have low single-unit drift compared to rigid electrodes.

(A) Representative drift traces of 3 recordings from monkey R PPC chamber using a Plexon V probe, Diagnostic Biochips 128 channel Deep Array probe, and our flexible array.

(B) Heatmap showing the drift activity of all Plexon, DBC, and our flexible array sessions from both monkeys O and R. Drift values are binned into 1 min windows.

(C) (Top) Box plot summarizing the mean absolute change in population spike-depth displacement across 5-minute windows for each session by probe type. Five-minute drift spikiness differed significantly across probe types (Kruskal-Wallis test, $\chi^2(3) = 35.14$, $p = 1.14 \times 10^{-7}$). Flexible arrays exhibited significantly lower 5-minute drift spikiness than Plexon probes (Wilcoxon rank-sum, $p = 3.10 \times 10^{-6}$) and DBC probes ($p = 1.89 \times 10^{-4}$). (Bottom) Box plot summarizing maximum displacement, defined as the peak-to-trough range of the median-centered population spike-depth drift trace for each session. Maximum displacement differed significantly across probe types (Kruskal-Wallis test, $\chi^2(3) = 36.06$, $p = 7.27 \times 10^{-8}$). Flexible arrays exhibited significantly lower maximum displacement than Plexon (Wilcoxon rank-sum, $p = 1.59 \times 10^{-6}$) and DBC probes ($p = 1.57 \times 10^{-5}$).

4. The Discussion claims that the microfabrication process can be scaled to "hundreds of channels with no additional cost or time," and that a double-sided 128-channel shank is "feasible." However, the current system already uses a 32-channel Intan headstage and a ZIF connector of non-negligible size. As channel count increases, cable bundle thickness, connector size, mechanical load on the microwire shuttle, and the complexity of synchronizing multi-shank telescopic systems all scale non-linearly.

We have revised this section to better discuss the design considerations that must be carefully considered when scaling microelectrode arrays.

"While the flexible arrays we used in this study have only 32 recording sites, the microfabrication process is scalable to larger channel counts. However, our results suggest that scaling comes with important design considerations, such as metal contact placement and overall shank width. The width can be partially mitigated using a multi-layer fabrication approach as shown in other studies (Fang et al., 2026, Fan et al., 2026, Böhler et al., 2023, Scholten et al., 2020, Le Floch et al., 2024). Importantly, increasing the electrode count also non-trivially increases the complexity and size of the I/O interface, which should be kept small to easily anchor to the recording chamber. Commercial, small-form factor headstages such as 128-channel devices offered by Plexon (HST/128DS Gen 1) and Cambridge Neurotech (Mini-Amp-128) are ideal. With these considerations, it is highly feasible to scale to a 128-channel shank that is compatible with our implantation approach and with a form factor suitable for regular use in academic NHP laboratories." (pg. 17)

5. The rigid probes compared against the flexible arrays were Plexon S/V-Probes and DBC Deep Array Probes. These differ drastically in mechanical stiffness and cross-sectional dimensions. the current state-of-the-art benchmark in NHP neurophysiology is Neuropixels. If possible, the drift comparison with data from Neuropixels should be added.

We performed recordings with Neuropixels, sorted single units, and performed a drift analysis as with the other arrays. This additional Neuropixels data was acquired from the PFC of two animals that differed from those in the original study (the animals used for the original submission were not available for further experiments). This means a direct comparison of drift between Neuropixels and our flexible electrodes must be interpreted carefully. We chose to analyze these data on an animal-by-animal basis to compare whether drift was consistently high across all recordings with rigid electrodes, and consistently low when flexible electrodes were used. Indeed, Neuropixels drift metrics were comparable to those observed in the other rigid probe classes and remained markedly elevated relative to flexible probes (Fig. S8, see below). We therefore present this data as a Supplementary Figure (see below) and made this point clear in the text and figure caption.

We made the following addition to the main text:

"To rule out the possibility that the reduction in probe-relative spike-depth drift was driven primarily by probe size rather than mechanical compliance, we performed additional recordings using Neuropixels probes, which have a substantially smaller form factor (70 µm shank width, 20 µm thickness) than the stainless-steel Plexon and DBC probes (~200 µm diameter). These recordings were obtained in two separate animals from the flexible, Plexon, and DBC datasets and should therefore be interpreted cautiously. Nevertheless, across animals, Neuropixels recordings exhibited drift metrics comparable to those observed with the rigid steel probes (Fig. S8, Table S1). In contrast, Neuropixels drift remained substantially greater than that observed with flexible arrays (maximum displacement, p = 0.00002; 5-minute drift velocity, p = 0.00007, Table S1).[DW9.1] To determine whether the reduced drift observed with flexible probes was

consistent across animals, we separated the drift calculation by subject. Flexible probe recordings consistently showed lower drift compared to rigid probes, while no significant differences in drift metrics were observed between animals (Fig. S8). Together, these findings suggest that the reduced drift observed with flexible arrays is not simply a consequence of a smaller probe profile but instead reflects the ability of mechanically compliant electrodes to better accommodate brain micromotion and improve recording stability." (pg. 12-13)

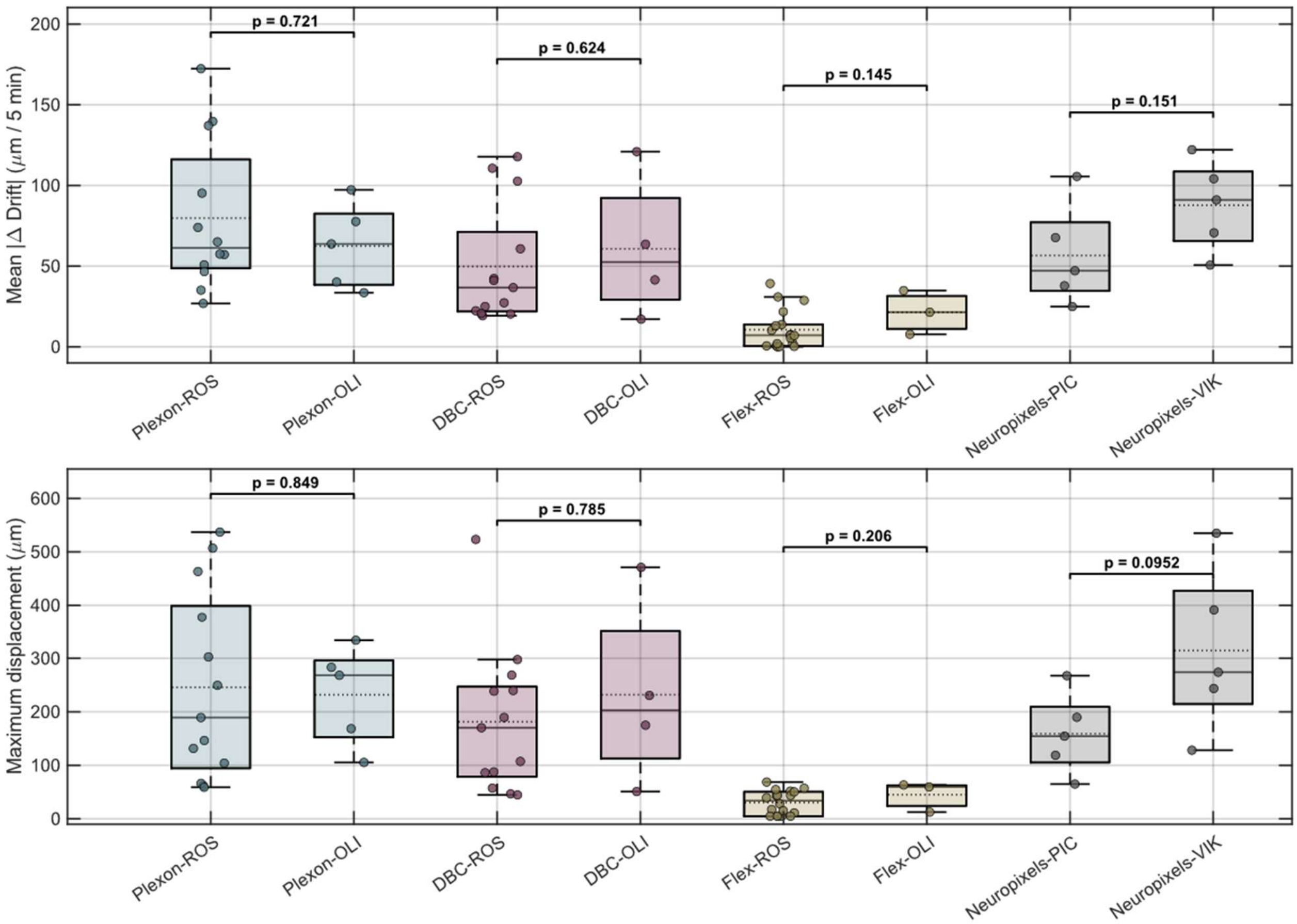

Figure S8. Flexible probes exhibit reduced drift across recording animals.
Animal separated distributions of probe drift metrics for Plexon, Diagnostic Biochips (DBC), flexible probes, and Neuropixels probes. Plexon, DBC, and flexible recordings were separated by animal identity (ROS and OLI), while Neuropixels recordings were separated by animal identity (PIC and VIK). (Top) Drift velocity over 5-minute intervals. (Bottom) Maximum displacement. Individual points represent recording sessions and box plots indicate the median and interquartile range. No significant differences in drift velocity or maximum displacement were observed between animals within any probe type (all within-probe animal comparisons, $p > 0.05$, Wilcoxon rank-sum tests). In contrast, data compiled for both animals for each probe type showed that drift metrics for all rigid probes significantly differ from flexible probes (Table S1).
